# Integrated Continuous Bioprocess Development for ACE-Inhibitory Peptide Production by *Lactobacillus helveticus* Strains in Membrane Bioreactor

**DOI:** 10.3389/fbioe.2020.585815

**Published:** 2020-09-25

**Authors:** Cyril Raveschot, Barbara Deracinois, Emmeline Bertrand, Christophe Flahaut, Marc Frémont, Djamel Drider, Pascal Dhulster, Benoit Cudennec, François Coutte

**Affiliations:** ^1^UMR Transfrontalière BioEcoAgro N°1158, Université de Lille, INRAE, Université de Liège, UPJV, YNCREA, Université d’Artois, Université du Littoral Côte d’Opale, ICV – Institut Charles Viollette, Lille, France; ^2^VF Bioscience, Loos-lez-Lille, France

**Keywords:** ACE-inhibitory peptides, fermented milk, gastrointestinal digestion, *Lactobacillus*, membrane bioreactor

## Abstract

Production of bioactive peptides (BAPs) by *Lactobacillus* species is a cost-effective approach compared to the use of purified enzymes. In this study, proteolytic *Lactobacillus helveticus* strains were used for milk fermentation to produce BAPs capable of inhibiting angiotensin converting enzyme (ACE). Fermented milks were produced in bioreactors using batch mode, and the resulting products showed significant ACE-inhibitory activities. However, the benefits of fermentation in terms of peptide composition and ACE-inhibitory activity were noticeably reduced when the samples (fermented milks and non-fermented controls) were subject to simulated gastrointestinal digestion (GID). Introducing an ultrafiltration step after fermentation allowed to prevent this effect of GID and restored the effect of fermentation. Furthermore, an integrated continuous process for peptide production was developed which led to a 3 fold increased peptide productivity compared to batch production. Using a membrane bioreactor allowed to generate and purify in a single step, an active ingredient for ACE inhibition.

## Introduction

Milk proteins are source of numerous bioactive peptides (BAPs). Proteins such as caseins and whey proteins have been used for the production of BAPs because inexpensive and safe sources are readily available (Wakai and Yanamoto, 2012). These peptides can be liberated from proteins upon gastrointestinal digestion (GID), enzymatic hydrolysis or microbial fermentation (Korhonen, 2009). Multiple biological activities, such as antihypertensive, antioxidant, opioid, immunostimulating or calcium binding activities have been associated with BAPs (Nongonierma and FitzGerald, 2015; Nielsen et al., 2017). The most extensively studied BAPs are those capable of inhibiting angiotensin converting enzyme (ACE). ACE catalyzes the conversion of angiotensin I into angiotensin II which acts as a vasoconstrictor leading to an elevated blood pressure; ACE inhibition therefore constitutes a major strategy to prevent hypertension (Guang et al., 2012; Wu et al., 2017).

The production of BAPs through fermentation is commonly conducted using lactic acid bacteria (LAB). Within this group, *Lactobacillus helveticus* (*L. helveticus*) is considered as a highly proteolytic species which could be used to generate specific peptide sequences by fermentation (Griffiths and Tellez, 2013; Raveschot et al., 2018). In particular, *L. helveticus* species are recognized for their capability to produce ACE-inhibitory peptides during fermentation (Wakai and Yanamoto, 2012; Elfahri et al., 2014; Ahtesh et al., 2018). The well-known ACE-inhibitory peptides Ile-Pro-Pro (IPP) and Val-Pro-Pro (VPP) are commonly found in milk fermented by various *L. helveticus* strains (Wakai and Yanamoto, 2012; Bakr Shori and Salihin Baba, 2015). Moreover, the peptide Ala-Ile-Pro-Pro-Lys-Lys-Asn-Gln-Asp (AIPPKKNQD) was also identified in milk fermented by the *L. helveticus* 130B4 strain (Shuang et al., 2008). These examples show that *L. helveticus* strains can be used to functionalize milk products, conferring health-beneficial properties to fermented milk thanks to the presence of BAPs (Marco et al., 2017).

Microbial fermentation is a cost effective method for BAPs production in comparison to the use of purified enzymes (Daliri et al., 2017). However, the industrial BAP production is still suffering from a lack of suitable large scale technologies (Wu et al., 2013; Agyei et al., 2016). Usually, fermented milks are prepared from batch fermentations without pH control generally resulting in yogurt-like, coagulated products (Li et al., 2017). In this case, optimization of BAPs production is difficult because of the absence of controlled conditions during the fermentation process. Furthermore, it was estimated that only 1–2% of milk proteins undergo proteolysis during fermentation by *Lactobacillus* species, resulting in low peptide productivity (Griffiths and Tellez, 2013). The use of bioreactors with controlled conditions during fermentation could improve peptide yields (Matar et al., 1996; Ahtesh et al., 2018). Among the different types of bioreactors, membrane bioreactors (MBR) operating in a continuous mode with total cell recycling using microfiltration, represents a suitable process to produce different biomolecules by fermentation (Coutte et al., 2017). Indeed, due to membrane retention, bacterial cells and non-hydrolyzed proteins can be concentrated in the bioreactor to undergo an advanced proteolysis during the process. Several studies used *Lactobacillus* strains in MBR for lactic acid production (Choudhury and Swaminathan, 2006; Xu et al., 2006; Probst et al., 2013; Taleghani et al., 2017; Tang et al., 2017) but to date, no report presented the use of MBR for BAPs production by Lactobacilli.

Another issue regarding the development of functional foods such as fermented milk is the impact of GID on the product. Indeed, proteolytic enzymes (pepsin, pancreatic enzymes, and intestinal brush barrier peptidases) present in the digestive tract will modify the peptide profile of fermented products following oral consumption. Consequently, the impact of GID on a given fermented product must be assessed to get a better prediction of the *in vivo* activities. GID increases the release of BAPs from fermented products, as they still contain proteins and large peptides that will be hydrolyzed during GID, generating small ACE-inhibitory peptides (Matar et al., 1996; Hernández-Ledesma et al., 2004; Jin et al., 2016).

The ultimate goal of this study was to develop a ready-to-use, BAPs-containing ingredient that would still present beneficial activity after GID. The targeted biological activity was ACE inhibition, which was assessed either through biochemical activity assay, or through peptide identification (mass spectrometry) followed by activity prediction using quantitative structure activity relationship (QSAR) modeling. To show the beneficial effect conferred by fermentation and to be closer to the real *in vivo* activities, the impact of a simulated GID was studied on the produced fermented milk. Finally, this work demonstrated the technical feasibility of an MBR-based continuous process for BAP production by *L. helveticus* strains.

## Materials and Methods

### Materials

Three *Lactobacillus* strains were used in this study: *L. helveticus* 45a, *L. helveticus* 49d, and *L. helveticus* 60b. These strains were previously isolated from different Mongolian dairy products based on their proteolytic abilities (Raveschot et al., 2020).

Maltodextrin (Glucidex IT 12) was obtained from Roquette (Lestrem, France). The fluorescent substrate for ACE (Abz-Gly-Phe(NO_2_)-Pro) was purchased from Bachem (Bubendorf, Switzerland). Finally, skim milk powder and all other reagents were purchased from Sigma-Aldrich (St. Louis, MO, United States).

### Batch Fermentation

#### Production of Fermented Milk

Each strain was taken from a frozen vial and transferred into De Man, Rogosa and Sharpe (MRS) medium (De Man et al., 1960) for 48 h growth at 37°C in anaerobic conditions. The resulting culture was used to inoculate a second preculture in a medium composed of skim milk (10%, w/v) and yeast extract (0.1%, w/v) (sterilized at 110°C for 30 min) for 24 h growth at 37°C in anaerobic conditions. This last step was repeated to obtain the final preculture used for batch fermentation.

Batch fermentation was conducted in a MiniBio 500 (Applikon Biotechnology, Delft, Netherlands) filled up with 300 mL of a skim milk medium supplemented with yeast extract as described above. The preculture was used to inoculate (10%, v/v) the medium with a starting optical density (OD_600nm_) of 0.3. Fermentation was conducted for 72 h at a constant temperature of 40°C with an agitation rate of 300 rpm in a final working volume of 330 mL. Dinitrogen was sparged continuously (20 mL.min^–1^) to maintain anaerobic conditions. The pH was set to 6 with H_3_PO_4_ (1 M) and NaOH (3 M) solutions. The skim milk fermentate was periodically sampled for determination of growth and peptide concentration. Experiments were performed in duplicate and mean values are presented. At the end of fermentation, the complete broth was centrifuged at 13,000 × *g* for 10 min and the supernatant was stored at −20°C for further analysis. In parallel, a control product (FCtl) was prepared, consisting of a non-inoculated medium but similarly incubated and centrifuged.

#### Bacterial Growth and Peptide Analysis

Samples taken from batch fermentation were analyzed for bacterial growth and peptide production using protocols previously described (Raveschot et al., 2020). Briefly, bacterial growth was determined by OD_600nm_ measurement after broth dilution in 2% ethylenediaminetetraacetic acid (EDTA), pH 12 solution. After centrifugation (13,000 × *g* for 10 min), the cell pellet was used for bacterial growth determination, and the supernatant was analyzed for protein and peptide contents using size exclusion chromatography (SEC) based on a fast protein liquid chromatography (FPLC) with a Superdex Peptide 10/300 GL column (10 × 300–310 mm, 13 μm, GE Healthcare, Little Chalfont, United Kingdom) coupled to an AKTA Protein Purification System (GE Healthcare, Little Chalfont, United Kingdom). Twenty-five microliters of sample were loaded, eluted in isocratic conditions with 30% acetonitrile (ACN), 0.1% trifluoroacetic acid (TFA) solution at a flow rate of 0.5 mL.min^–1^ for 60 min and monitored at 214 nm. The column was calibrated using compounds of known molecular weight (Glutathione 307.3 Da, Vitamin B12, 1,355 Da, Aprotinin, 6,500 Da, Cytochrome C, 12,400 Da and Albumin, 60 kDa for the dead volume). Protein and peptide contents, and peptide molecular weight distribution in each sample were estimated through chromatogram integration. Two molecular weight ranges were defined, the first corresponding to the high molecular weight proteins/peptides (HMWPs) with an apparent molecular weight higher than 1,700 Da, and the second, to the low molecular weight peptides (LMWPs) with an apparent molecular weight lower than 1,700 Da. The content estimation for each range was expressed as percentage of the area under the curve (%HMWPs and %LMWPs). Finally, peptide concentration in each sample was assessed after protein precipitation with trichloroacetic acid (TCA, 1% final concentration) using the Folin-Ciocalteu reagent (Lowry et al., 1951; McSweeney and Fox, 1997). A peptide digest standard (Peptide digest assay standard, Thermo Fisher Scientific, Waltham, MA, United States) was used for peptide quantification.

#### Peptide Separation by Ultrafiltration

After batch fermentation, the centrifugated broth was either kept intact (F Raw) or submitted to an ultrafiltration step for peptide separation. The membrane used was a Hydrosart cartridge (Sartorius, Göttingen, Germany) of 0.1 m^2^ with a 10 kDa molecular weight cut off. The resulting permeate from ultrafiltration (F UFP) containing molecules lower than 10 kDa was dried by centrifugal evaporation (miVac Centrifugal Vacuum Concentrators, Gene Vac, Ipswich, United Kingdom) at 40°C to 10% of the initial volume.

### Simulated Gastrointestinal Digestion

The products obtained from batch fermentation (F Raw and F UFP) were subjected to an *in vitro* static simulated gastrointestinal digestion (SGID). Gastric and intestinal steps of the GID process were successively simulated by adding different fluids to the fermented product. The protocol used and fluids composition were described by Caron et al. (2015). Briefly, 20 mL of fermented product containing 100 g.L^–1^ of dry matter were mixed with 24 mL of gastric fluid containing 37.5 mg of porcine pepsin (E:S ratio = 1/40). The reaction was conducted for 2 h at 37°C with a constant pH of 2.5–3. After the gastric phase, the intestinal step was simulated by the addition of 12 mL of bile solution, 24 mL of intestinal fluid containing 16.65 mg of porcine pancreatic enzyme (E:S ratio = 1/50), and 4 mL of NaHCO_3_ (1 M). The reaction was conducted for 2 h at 37°C with a constant pH of 7–7.5. Samples were taken at the end of the gastric and intestinal steps (called G2 and I2, respectively), inactivated by heat treatment and analyzed for peptide content by Folin-Ciocalteau reagent and SEC as described above. For each fermented product (F Raw and F UFP), experiments were performed in triplicate.

### ACE Inhibition Analysis

#### Biochemical Test

The biochemical test used to evaluate the ACE-inhibitory activity of fermented products was described by Sentandreu and Toldrá (2006). Briefly, samples, ACE and its substrate were prepared in a 150 mM Tris–HCl, pH 8.3 buffer. The inhibition reaction was performed in a final volume of 300 μL containing 50 μL of samples (or buffer for negative control), 50 μL of ACE solution (0.05 U.mL^–1^) and 200 μL of ACE substrate (Abz-Gly-Phe(NO_2_)-Pro) (0.45 mM). A control without ACE was also used where the enzyme was replaced by buffer solution. The fluorescence of the enzymatic reaction product substrate was monitored every 2 min for 1 h at 37°C in a spectrofluorometer (Xenius XC, Safas Monaco, Monaco, France) with excitation and emission wavelengths of 365 and 415 nm, respectively. The inhibition percentage toward ACE was defined as the percentage of ACE activity inhibited by a given concentration of the fermented products compared to the negative control. For each product, the concentration required to cause 50% of inhibition of ACE activity (IC_50_) was determined by plotting the inhibition percentage as a function of sample final concentration natural logarithmic. IC_50_ were expressed in mg.mL^–1^ of dry matter. Experiments were performed in triplicate.

#### Prediction of ACE-Inhibitory Activity

Peptides contained in the raw fermented products before and after SGID (F Raw and F Raw I2, respectively) were purified and identified by mass spectrometry (UPLC-ESI-qTOF-MS/MS) as described previously (Raveschot et al., 2020). Peptides were purified on Bond Elut C_18_ microcolumns (Agilent Technologies, Santa Clara, CA, United States) and dried by centrifugal evaporation (miVac Centrifugal Vacuum Concentrators, Gene Vac, Ipswich, United Kingdom) for 2 h at 40°C. Dried peptides were redissolved in 100 μL H_2_O, 0.1% TFA. Ten microliters were chromatographed on an ACQUITY UPLC system (Waters, Manchester, United Kingdom) using a C_18_-Kinetex column (150 × 4.6 mm, 2.6 μm 100 Å, Phenomenex, Torrance, CA, United States), a linear gradient of ACN containing 0.1% FA and a flow rate of 500 μL.min^–1^. The HPLC eluent was directly electrosprayed from the column end at an applied voltage of 3 kV, using a desolvation gas (dinitrogen) flow of 600 L.h^–1^, a nebulizer gas flow of 2.5 bar and desolvation temperature of 300°C, respectively. The chromatography device was coupled to SYNAPT-G2-Si mass spectrometer (Waters, Manchester, United Kingdom). MS analysis was performed in sensitivity, positive ions and data dependent analysis (DDA) modes and MS data were collected in the 100–2,000 m/z range with a scan time of 0.2 s. A maximum of 15 precursor ions with an intensity threshold of 10,000 counts were selected for ion trap collision-induced dissociation (CID) fragmentation and subjected to a collision energy ramping from 8 to 9 V for low mass and 40 to 90 V for high mass. The MS/MS spectra were recorded on the 100–2,000 m/z range with a scan time of 0.1 s. Peaks were analyzed using Mass Lynx software (4.1. version, Waters, Manchester, United Kingdom).

The 3D-maps of LC-MS/MS signals were analyzed using Progenesis QI for proteomics software (4.0. version, Nonlinear Dynamics, Manchester, United Kingdom) by principal component analysis (PCA). Database searches via PEAKS Studio 7.0 (Bioinformatics Solutions Inc., Waterloo, ON, Canada) were performed using the UniProt databases (May 15, 2017) restricted to *Bos taurus* organism. A mass tolerance of 35 ppm and three missing cleavage sites, no specific enzyme, variable methionine oxidation and an MS/MS tolerance of 0.2 Da were allowed. No fixed modification was used for the peptide identifications. The relevance of protein and peptide identities was judged according to their identification generated by PEAKS Studio 7.0 (*p* < 0.05) and a false discovery rate <to 1%. Experiments were performed in triplicate.

For each fermented product, the prediction of ACE-inhibitory activity was only performed on peptides identified in all three technical repetitions of the LC-MS/MS analysis. Prediction of ACE-inhibitory activity was performed using a QSAR model developed by Pripp et al. (2004). This model predicts IC_50_ for each peptide based on the last two amino acids found at the C-terminal position. QSAR modeling was performed using Microsoft Excel software (2013 version). Predicted IC_50_ were then categorized and frequencies for each range of IC_50_ were calculated. Results were expressed as the percentage of the total number of peptides in a given fermented product.

### Continuous Process for Peptide Production

Continuous culture was performed without total cell recycling in a classical bioreactor. Fermentations were performed in a MiniBio 500 filled with 300 mL of medium. Firstly, strain was cultivated in a classical batch mode for 24 h in anaerobic conditions (dinitrogen sparged) at 40°C with a pH maintained at 6 and agitation rate of 300 rpm. After 24 h, the fermenter was continuously fed at a dilution rate of 0.1 h^–1^ (30 mL.h^–1^) and broth was withdrawn from the fermenter at the same flow rate.

For each experiment, the broth was periodically sampled for determination of growth, peptide and carbohydrate (lactose and lactic acid) concentrations. Experiments were performed in duplicate and mean values were presented.

### Continuous Process for Peptide Production in Membrane Bioreactor

Continuous fermentation was also performed in a membrane bioreactor (MBR) (Applikon Biotechnology, Delft, Netherlands) containing 3 L of the previously described skim milk medium. The bioreactor was coupled with a 3,600 cm^2^ hollow fiber membrane with 0.2 μm of pore size (GE Healthcare, Little Chalfont, United Kingdom). Batch phase of culture was performed as described above. Hereafter, during the continuous phase, a peristaltic pump was used for broth circulation from the bioreactor to the filtration membrane at a flow rate of 1.1 L.min^–1^. The fermenter was continuously fed at the same dilution rate of 0.1 h^–1^ (0.3 L.h^–1^) with fresh medium. In order to keep the fermenter volume constant, permeate of the microfiltration was withdrawn at a same flow rate as the feeding. The fermented product containing the produced peptides was collected in a tank and stored at −20°C for further analysis. For each experiment, the broth was periodically sampled for determination of growth, peptide and carbohydrate (lactose and lactic acid) concentrations. Experiments were performed in duplicate and mean values are presented.

### Analytical Methods for Fermentation Monitoring

Strain growth and peptide quantification by the Folin-Ciocalteu reagent were performed on samples as described in the “Bacterial Growth and Peptide Analysis” section. Carbohydrates analysis were performed by high performance liquid chromatography (HPLC) using a Fast Fruit Juice (50 Å, 7 μm, 7.8 × 50 mm, Waters, Manchester, United Kingdom) column coupled to a Spectra SYSTEM (Thermo Electron Corporation, San Jose, CA, United States). Briefly, 20 μL of samples were chromatographed in isocratic condition with a 0.05 M H_3_PO_4_ solution at a flow rate of 0.8 mL.min^–1^ for 10 min. Eluted molecules were detected by a refractometer (Spectra SYSTEM RI-150, Thermo Electron Corporation, San Jose, CA, United States).

### Spray Drying of the Fermented Product and Biological Activity of the Resulting Ingredient

Atomization of the fermented product (which corresponds to the total permeate of the microfiltration) obtained from the MBR was performed with a Mini atomizer B-290 (Buchi, Rungis, France). A maltodextrin powder (Glucidex IT 12) was used as a charge agent at 0, 5, 10 or 20% (w/v). For each experiment, 200 mL of fermented product at room temperature (22°C) were pulverized at a flow rate of 8 mL.min^–1^ and dried by air set at 130°C. Characteristics of the inlet air were analyzed using a psychrometer and were 22°C with 64% of humidity (dew temperature of 15.5°C and wet bulb temperature of 17.9°C). The outlet temperature of the final dried product was about 68°C. Atomization yields were calculated according to the dry matter collected after spray drying process on the theoretical dry matter before atomization. Experiments were performed in triplicate.

Finally, the ACE-inhibitory activity of the fermented product before and after atomization was evaluated by a biochemical test as described in the “Biochemical Test” section.

### Statistical Analysis

All statistical analysis were performed using one-way ANOVA and Tukey’s *post-hoc* test for pairwise comparison. Differences between means were considered significant when *p* value < 0.05. Statistical analyses were performed on R software (R core team, 2016, Vienna, Austria).

## Results and Discussion

### Strain Growth and Peptide Production During Batch Fermentation

**FIGURE 1:**
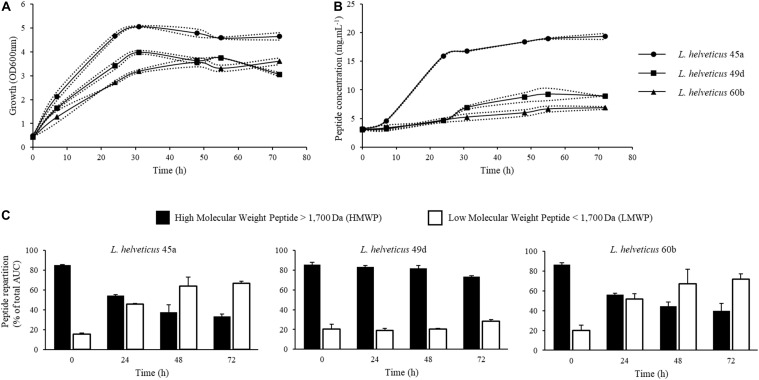
Kinetics of growth, peptide concentration and integration of chromatograms from size exclusion chromatography (SEC) of peptides during batch cultures in bioreactor of *L. helveticus* 45a, *L. helveticus* 49d, and *L. helveticus* 60b. **(A)** Strain growth was monitored by optical density determination at 600 nm. **(B)** Peptide concentration in bioreactor was determined by Folin-Ciocalteu reagent. **(C)** SEC profile integration of peptides obtained on a Superdex Peptide 10/300 GL column. Dotted lines represent the experimental data and full lines represent the mean from two independent experiments.

Batch fermentations were conducted in order to produce fermented milk products from three strains of *L. helveticus*. Strains were inoculated at an initial OD_600nm_ of 0.3 (Figure 1A). During the exponential phase, the specific growth rate (μ) was about 0.09 h^–1^ for *L. helveticus* 45a, 0.07 h^–1^ for *L. helveticus* 49d, and 0.08 h^–1^ for *L. helveticus* 60b. After 31 h of culture, cell concentrations remained constant for the rest of the fermentation process. The highest biomass concentration was observed for *L. helveticus* 45a strain, reaching an OD_600nm_ of about 5. The two other strains reached an OD_600nm_ of 3–4. The initial peptide concentration in the skim milk medium was 3.12 g.L^–1^ right after inoculation (Figure 1B), these peptides originating most probably from the inoculum. Peptide concentration increased with time proportionally to the strain growth, with a particularly important production during the first 31 h of culture with the strain *L. helveticus* 45a. Thereafter, until the end of the fermentation, peptide concentrations increased slowly or remained constant depending on the strain considered. The highest concentration was observed with the *L. helveticus* 45a strain, reaching 19.33 g.L^–1^ after 72 h of culture. For strains *L. helveticus* 49d and 60b, peptide concentrations reached values of 6.87 and 8.91 g.L^–1^, respectively. In order to follow protein hydrolysis during fermentation, samples from the bioreactor were analyzed by SEC (Figure 1C). Molecular weight repartitions at the beginning of the culture were 85–86% for HMWPs and 15–20% for LMWPs. As a result of protein hydrolysis by *Lactobacillus* strains, the proportion of LMWPs increased over time during fermentation. The homofermentative profile of *L. helveticus* leads to an important production of lactic acid from lactose and consequently a high acidification capacity (Hebert et al., 2000). In this study, a pH value of 6 was chosen to prevent protein precipitation in the medium, while being close to the optimal pH for *Lactobacillus* proteinase activity (Fira et al., 2001). This approach has been used by Matar et al. (1996) for *L. helveticus* L89 strain. Indeed, the bacterial growth was not affected by a pH-controlled vs. a non-controlled one, but the peptide production was significantly improved in a pH-controlled medium.

After fermentation, products were centrifuged for bacterial cell elimination. The supernatants were either used directly (F Raw) or subjected to an ultrafiltration step in order to obtain permeates without proteins (F UFP). Moreover, a control product consisting in a non-fermented milk was included in the study.

### Changes in the Peptide Content of Fermented Products During SGID

**FIGURE 2:**
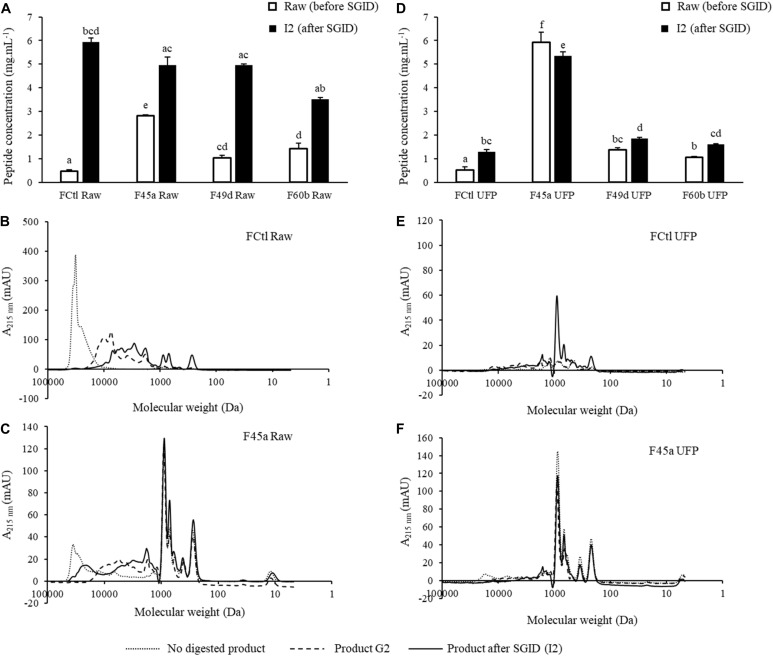
Evolution of peptide concentrations and size exclusion chromatography (SEC) profiling during *in vitro* simulated gastrointestinal digestion (SGID) of fermented and control products. Products were submitted either in their raw (Raw) or in their ultrafiltrated forms (UFP). **(A,D)** Evolution of peptide concentrations determined by the Folin-Ciocalteu reagent before and after SGID of the raw and the UFP products. Data were represented as mean ± SD of three independent experiments. Statistical analysis using one way ANOVA and Tukey’s *post-hoc* test. Mean values in the same graph without a common letter differ significantly (*p* value < 0.05). **(B,C,E,F)** SEC profiles of peptides from the FCtl Raw, the F45a Raw, the FCtl UFP, and the F45a UFP products before (not digested), at the end of the gastric step (G2) and at the end of SGID (I2). SEC profiles were obtained on a Superdex 10/300 GL column.

A SGID was performed on fermented products to study the impact of gastrointestinal proteolysis on peptide content and ACE-inhibitory activity. Indeed, GI enzymes can further degrade partially hydrolyzed proteins in fermented products, generating new, smaller peptides which are potentially bioactive (Caron et al., 2016; Daliri et al., 2017; Lamothe et al., 2017; Sanchón et al., 2018). Peptide quantification was performed before and after SGID; molecular weight repartition was analyzed by SEC (Figure 2 and Table 1). Notably, raw fermented products showed an important increase of their peptide content after SGID (Figure 2A), with concentrations increasing from 2 to 12 fold. These observations were confirmed by SEC analyses : the control product (FCtl Raw) showed a significant modification of its apparent peptide molecular weight distribution, with a complete hydrolysis of high molecular weight proteins after the intestinal phase (Figure 2B and Table 1). A similar observation was made for the F45a Raw product (Figure 2C and Table 1) although in this case, because of bacterial fermentation, the proportion of HMWPs before GID was lower than in the control sample. These results are consistent with previous studies showing that milk proteins are very efficiently digested upon SGID or passage through human jejunum (Sanchón et al., 2018). For UFP products, modifications in peptide content before and after SGID were much smaller (Figure 2D and Table 1), except in the control product (FCtl UFP I2) where a notable increase was observed, probably resulting from of the auto-hydrolysis of GI enzymes. Regarding molecular weight distribution, SGID of F UFP products did not lead to major changes. SEC analysis showed the efficacy of the ultrafiltration step with an absence of molecules higher than 10 kDa (Figures 2E,F and Table 1). Altogether, these results suggest that ultrafiltered products are much less sensitive to alterations by GI enzymes, and therefore that biological activities of these products may be maintained even in an *in vivo* setting. It has already been reported the tripeptides Ile-Pro-Pro and Val-Pro-Pro produced by some *L. helveticus* strains, are not affected by SGID (Ohsawa et al., 2008).

**TABLE 1:** SEC profile integration of peptides from each fermented and control product before (no digested product), during (product G2), and after GID (product after GID, I2).

F Ctl Raw					F Ctl UFP				
	
	%HMWP	SD	%LMWP	SD		%HMWP	SD	%LMWP	SD
No digested product	97.9	0.4	2.1	0.4	No digested product	10.2	0.7	89.8	0.7
Product G2	76.2	1.6	23.8	1.6	Product G2	33.2	2.2	66.8	2.2
Product after GID (I2)	43.3	1.4	56.7	1.4	Product after GID (I2)	4.1	3.6	95.9	3.6

**F 45a Raw**					**F 45a UFP**				

No digested product	92.0	0.7	8.0	0.7	No digested product	10.7	0.5	89.3	0.5
Product G2	38.5	1.6	61.5	1.6	Product G2	4.6	3.6	95.4	3.6
Product after GID (I2)	19.4	3.2	80.6	3.2	Product after GID (I2)	1.0	0.5	99.0	0.5

**F 49d Raw**					**F 49d UFP**				

No digested product	86.1	1.7	13.9	1.7	No digested product	3.3	2.9	96.7	2.9
Product G2	67.5	3.3	32.5	3.3	Product G2	10.8	9.5	89.2	9.5
Product after GID (I2)	34.7	0.9	65.3	0.9	Product after GID (I2)	0.2	0.2	99.8	0.2

**F 60b Raw**					**F 60b UFP**				

No digested product	80.7	4.7	19.3	4.7	No digested product	2.5	1.3	97.5	1.3
Product G2	58.5	4.5	41.5	4.5	Product G2	8.6	8.5	91.4	8.5
Product after GID (I2)	30.7	1.8	69.3	1.8	Product after GID (I2)	1.1	0.9	98.9	0.9

*Products were tested either in a raw form (F Raw) or either after an ultrafiltration step (F UFP). SEC profiles were obtained on a Superdex 10/300 GL column. Two molecular weight ranges were defined, the first corresponding to the high molecular weight proteins/peptides (HMWP) with an apparent molecular weight higher than 1,700 Da, and the second, to the low molecular weight peptides (LMWP) with an apparent molecular weight lower than 1,700 Da. The content estimation for each range was expressed as percentage of the area under the curve (%HMWP and %LMWP).*

### ACE Inhibition Activities of the Produced Hydrolysates

**FIGURE 3:**
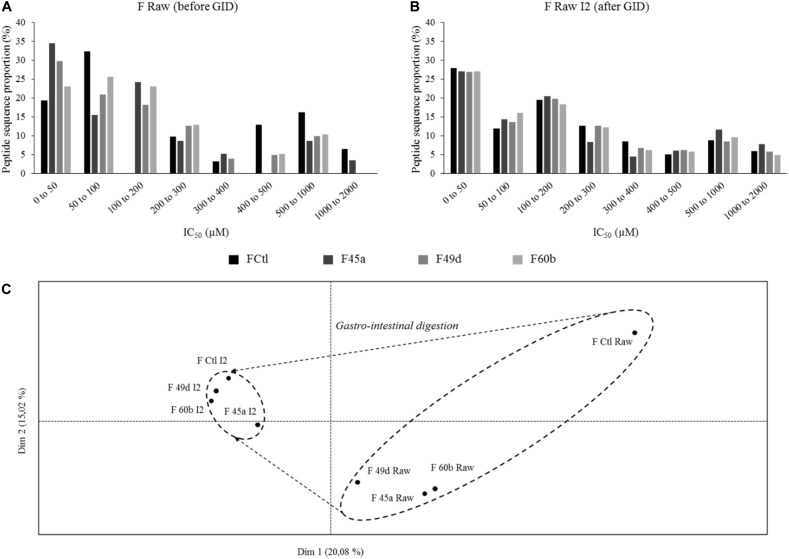
Predicted IC50 toward angiotensin converting enzyme (ACE) inhibition repartition of identified peptides and principal component analysis (PCA) of the 3D-maps obtained from mass spectrometry (LC-MS/MS) analysis of peptides extracted from the fermented and control products. IC50 were predicted using a quantitative structure activity relationship (QSAR) model and peptide proportions in different ranges of IC50 were calculated for (from dark to light) the control product (FCtl) and the products obtained by fermentation with *L. helveticus* 45a (F45a), *L. helveticus* 49d (F49d), and *L. helveticus* 60b (F60b) strains. **(A)** Predicted IC50 repartition from raw products. **(B)** Predicted IC50 repartition from products submitted to a simulated gastro-intestinal digestion (SGID). **(C)** PCA of 3D-maps obtained from LC-MS/MS analysis of peptides extracted from the raw products before (Raw) and at the end of the SGID (I2).

Angiotensin converting enzyme-inhibitory activity in *Lactobacillus* fermented products was already reported (Fernandez et al., 2017; Georgalaki et al., 2017; Kliche et al., 2017; Solanki et al., 2017; Alhaj et al., 2018), and numerous ACE-inhibitory peptides have been identified (Pihlanto, 2013; Li et al., 2017). In this study, ACE-inhibitory activity was assessed by a biochemical test performed on samples, and predicted using a QSAR model on peptides identified by LC-MS/MS. 85–317 peptide sequences were identified in the fermented samples (F Raw), and 283–511 sequences in the products which had undergone simulated GI digestion (F Raw I2). Database searches showed that none of these sequences came from yeasts or *L. helveticus* proteomes. Peptides were considered to display a relevant ACE-inhibitory activity when the predicted IC_50_ was lower than 200 μM. As an example, our QSAR model predicted an IC_50_ of 26.85 μM for the well-known ACE inhibitors IPP and VPP. Predicted IC_50_ varied widely in the fermented products, with values ranging from 5.8 to 2000 μM (Figure 3A). 19% of the peptides in non-fermented milk (FCtl Raw) had a predicted IC_50_ lower than 50 μM, 32% between 50 and 100 μM and 0% between 100 and 200 μM. This repartition profile corresponds to a basal level of inhibitory peptides found in raw, untreated milk. By comparison, the fermented products showed higher proportions (23–34%) of peptides with a predicted IC_50_ lower than 50 μM. Fermentation therefore led to an enrichment of the samples in peptides with high ACE-inhibitory activity. However, applying a simulated GID after fermentation led to a complete loss fermentation’s benefits : the proportion of peptides with a predicted IC_50_ lower than 50 μM became virtually similar (between 27 and 27.89%) in the non-fermented control, and in the three fermented products (Figure 3B). To confirm these observations, peptide heterogeneity between samples was evaluated by conducting a PCA on 3D-maps of LC-MS/MS signals (Figure 3C). The principal components, named Dim1 and Dim2, explained 20.08 and 15.02% of the total variance, respectively. F Raw samples were scattered along the positive side of Dim1 whereas the digested products (F Raw I2) were grouped on the negative side. Samples treated by SGID (including the non-fermented control) were grouped together as a small, homogeneous cluster whereas for raw, undigested samples, the non-fermented control was clearly separated from the fermented products. SGID therefore appears to erase the differences between non-fermented control and fermented samples, in terms of both peptide composition and predicted ACE-inhibitory activity.

Angiotensin converting enzyme-inhibitory activity was also evaluated *in vitro* using a biochemical assay. Table 2 shows the IC_50_ determined for the fermented products and after their treatment by ultrafiltration, SGID or both. All raw fermented products presented lower IC_50_ than the non-fermented milk : IC_50_ for non-fermented milk was 12.76 mg.mL^–1^, whereas the IC_50_ for the two most potent products, F45a and F49d, were 0.57 and 0.76 mg.mL^–1^, respectively. After SGID (F Raw I2), a decrease of IC_50_ was observed in all products (IC_50_ values ranging from 0.25 to 0.42 mg.mL^–1^) but there were no more statistically significant differences between the samples. These results are in accordance with the bioactivities predicted using the QSAR model, and with the PCA analysis of peptide homogeneity; in particular, the correlation between the proportion of peptides with predicted high inhibitory capacity and the results of the biochemical test demonstrate the relevance of our QSAR model. An important observation, though, is that performing SGID on samples totally erases the differences which existed initially between the fermented samples and the non-fermented control, thereby causing a loss of the fermentation’s benefit. According to these observations, the consumption of fermented milk is no more beneficial than the consumption of non-fermented milk.

**TABLE 2:** IC_50_ toward ACE inhibition determined *in vitro* from each fermented and control product.

F Raw			F UFP		
	
	IC_50_ (mg.mL^–1^)	SD		IC_50_ (mg.mL^–1^)	SD
FCtl	12.76^*c*^	0.07	FCtl	8.38^b^	1.78
F45a	0.57^a^	0.03	F45a	0.47^a^	0.03
F49d	0.76^a^	0.03	F49d	1.76^a^	0.1
F60b	4.22^b^	0.09	F60b	2.11^a^	0.46

**F Raw after GID (F Raw I2)**			**F UFP after GID (F UFP I2)**		

FCtl	0.32^a^	0.15	FCtl	2.13^b^	0.4
F45a	0.38^a^	0.08	F45a	0.56^a^	0.05
F49d	0.25^a^	0.04	F49d	1.97^b^	0.64
F60b	0.42^a^	0.14	F60b	3.3^b^	0.44

*Products were tested either in a raw form (F Raw) or either after an ultrafiltration step (F UFP). These products were also tested after a SGID (I2). The statistical analysis were performed using one-way ANOVA and Tukey’s *post-hoc* test. Mean values in the same part without a common letter differ significantly (*p* value < 0.05).*

To prevent the impact of SGID on the biological activity, an ultrafiltration step was conducted on the fermented products to remove the proteins that had not been hydrolyzed during the fermentation process. The distribution of IC_50_ from these ultrafiltrated products (F UFP) showed the same tendency as the one observed for raw products (Table 2). An IC_50_ of 8.38 mg.mL^–1^ was reported for the control (FCtl UFP), compared to 2.11 to 0.47 mg.mL^–1^ for the fermented products, with the F45a UFP being the most potent product. After SGID, the IC_50_ of the non-fermented control (FCtl UFP I2) decreased to 2.13 mg.mL^–1^. The F49d and F60b UFP I2 presented similar IC_50_ of 1.97 and 3.3 mg.mL^–1^, respectively. Finally, the IC_50_ value for the F45a UFP I2 was four times lower than the control one, at 0.56 mg.mL^–1^ (statistically significant difference). Therefore, the impact of fermentation was maintained even after SGID in milk fermented with the *L. helveticus* 45a strain.

Altogether, these results demonstrate the ability of the strain *L. helveticus* 45a to produce ACE-inhibitory peptides by milk fermentation. Nevertheless, a peptide separation step was required to obtain a significant improvement of the biological activity in comparison to a non-fermented milk. A fragmentation strategy could now be used to identify peptides generated by *L. helveticus* 45a, and involved in ACE inhibition (Sharma et al., 2011).

### Continuous Fermentation in Membrane Bioreactor

The above results allow two important conclusions : (i) fermentations conducted in batch mode are only partial and leave large amounts of undigested proteins, which during GID can be further hydrolyzed to bring peptides of interest; (ii) an ultrafiltration step on the culture broth allows to standardize the end-product with respect to the GID by keeping only the most active small peptides produced by *Lactobacillus* strains. Based on these results, an integrated continuous process for peptide production was developed using the *L. helveticus* 45a strain. One of the limitations of the use of Lactobacilli for peptide production is the lack of efficient industrial processes (Agyei et al., 2016). Experiments were performed either in a classical bioreactor (CBR) or in a membrane cell-recycle bioreactor (MBR) consisting in a bioreactor coupled to a microfiltration membrane (0.2 μm). The use of an MBR presents the advantage to simultaneously, in a single step, produce and purify an active ingredient. Additionally, high peptide yield can be obtained because of the concentration of proteins and bacterial cells in the bioreactor.

**FIGURE 4:**
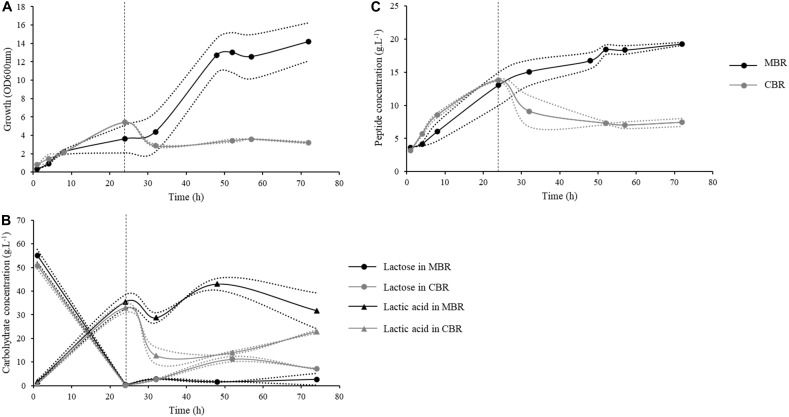
Kinetics of growth, peptide concentrations and carbohydrate concentrations during continuous processes conducted in a classical (CBR, gray) or a membrane bioreactor (MBR, black) with the *L. helveticus* 45a strain. The first 24 h of culture were conducted in batch mode, then bioreactors were fed continuously at a dilution factor of 0.1 h^–1^. **(A)** Strain growth was monitored by optical density determination at 600 nm. **(B)** Peptide concentration in bioreactor were determined by Folin-Ciocalteu reagent. **(C)** Lactose (circle) and lactic acid (triangle) concentrations were determined by high performance liquid chromatography (HPLC) using a Fast Fruit Juice column. Dotted lines represent the experimental data and full lines represent the mean from two independent experiments.

Bacterial strains were cultured in the same conditions as for batch fermentation. The initial OD_600nm_ at the beginning of the culture was 0.3 (Figure 4A). During the first 24 h, fermentations were conducted in batch mode, and bacterial concentration reached an OD_600nm_ mean of 4.5. After switching to the continuous mode, the bacterial concentration in CBR decreased to reach an OD_600nm_ of 2.7 after 32 h of culture, before stabilizing for the rest of the culture at an OD_600nm_ of about 3. Conversely to CBR, the bacterial concentration in MBR showed a strong increase after 24 h of continuous mode, reaching an OD_600*nm*_ of 12.7, as a result of bacterial retention by the membrane. Bacterial growth then stabilized, the OD_600nm_ finally reaching a value of 14.4 at the end of the culture. Substrate availability during fermentation was evaluated by quantification of lactose and lactic acid. The initial lactose concentration was about 52 g.L^–1^ at the beginning of the culture (Figure 4B). During the batch phase, an important decrease of lactose concentration was observed with only 0.1 g.L^–1^ of lactose left after 24 h. Consistently, lactic acid concentrations increased from 0.8 to 34 g.L^–1^. During the continuous phase, lactose concentration increased in the CBR, reaching 11 g.L^–1^ after 52 h of culture and then remained stable until the end of fermentation. Lactic acid concentration decreased at the beginning of the continuous phase to reach a concentration of 12 g.L^–1^ after 32 h of culture. A continuous increase of lactic acid concentration was then observed for the rest of the culture (22 g.L^–1^ at the end of the fermentation). In the MBR, lactose concentration remained constant during the continuous phase, reaching 1.6 g.L^–1^ at 48 h of culture suggesting that lactose supplied during this phase was instantly consumed by the bacterial population. Lactic acid concentration increased at the beginning of the continuous phase, following the bacterial growth and reached 43 g.L^–1^ after 48 h of culture. A stabilization of lactic acid concentration was then observed for the rest of the fermentation. Lactic acid can inhibit the growth of *Lactobacillus* strains (Aguirre-Ezkauriatza et al., 2010), the important concentration of lactic acid found after 48 h of culture could therefore explain the growth limitation observed in MBR. Moreover, the dilution rate applied during the continuous phase (0.1 h^–1^, flow rate of 5 mL.min^–1^) represented also a limitation for bacterial growth probably due to a lack of lactose supply in the bioreactor. Indeed, during the initial 24 h batch phase, bacterial cells consumed a large part of the lactose initially found in milk; lactose supply during the continuous phase was then insufficient, resulting in growth limitation after 48 h of culture. Regarding peptide production, the initial peptide concentration was equal to 3 g.L^–1^, this concentration increased to 14 g.L^–1^ at the end of the batch phase (Figure 4C). In CBR, peptide concentration decreased after 52 h of culture to reach 7 g.L^–1^ and then remained constant until the end of the fermentation process. Regarding the MBR, peptide concentration increased slightly during the continuous phase in comparison to the batch mode, reaching 19 g.L^–1^ after 72 h of culture. In this bioreactor, peptides were separated from the medium by the microfiltration membrane. However, an important part of the peptides remained in the bioreactor. After 72 h of culture, peptide quantities in the bioreactor and in the product tank were equal to 57.9 and 155.2 g, respectively. This clearly indicates a phenomenon of peptide retention by the microfiltration membrane during the process. Retention of non-casein nitrogen during milk microfiltration was observed previously (Boiani et al., 2017; Renhe and Corredig, 2018) probably due to interactions with caseins and calcium-phosphate clusters (Cross et al., 2005).

Peptide productivities of the different processes (batch, continuous in CBR and in MBR) used in this study were compared (Table 3). The productivity at the stationary phase (72 h of culture) was higher in the MBR. Similarly, the mean productivity in MBR was equal to 0.27 g.L^–1^.h^–1^, vs. 0.1 g.L^–1^.h^–1^ in the CBR, showing the interest of MBR approach for peptide production. Moreover, fermentation conducted in batch mode showed an equivalent productivity (0.33 g.L^–1^.h^–1^) in comparison to fermentation in MBR. Peptide productivities were also calculated as a function of the bacterial biomass, leading to the determination of specific productivities. The continuous process in CBR showed a specific productivity of 7.13 g.g^–1^ which is close to that obtained in batch mode (9.99 g.g^–1^); continuous process in MBR, however, presented a much higher productivity (15.8 g.g^–1^). The instantaneous productivity in CBR was equal to 0.74 g.L^–1^.h^–1^, compared to 1.93 g.L^–1^.h^–1^ in the MBR, showing the interest for using this type of bioreactor in a continuous process. Concomitantly, the instantaneous productivity in batch mode was calculated at the maximum peptide production time (between 7 and 24 h of culture) and was equal to 0.67 g.L^–1^.h^–1^. Finally, the use of a continuous process in MBR increased by almost 3-fold the instantaneous peptide productivity in comparison to the batch process. Moreover, it was calculated that 5.3 batch fermentations (whose preparation is time consuming) would be required to obtain the same peptide quantity than the one produced with only one continuous process in MBR. An important lactic acid production was observed during this process; actually, *Lactobacillus* cultures were generally conducted in MBR to study lactic acid production from various nutrient sources (Choudhury and Swaminathan, 2006; Xu et al., 2006; Probst et al., 2013; Taleghani et al., 2017; Tang et al., 2017). Further optimizations, particularly on the dilution rate applied during the continuous phase, could lead to a further improvement of peptide productivities by this process.

**TABLE 3:** Mean peptide concentration and peptide productivities from the different processes used in this study.

	Batch (48 h)	Continuous (CBR, 72 h)	Continuous (MBR, 72 h)
Mean peptide concentration (g.L^–1^)	15.9 (±0.1)	7.43 (±0.8)	19.3 (±0.3)
Mean productivity (g.L^–1^.h^–1^)	0.33 (±0.01)	0.103 (±0.02)	0.27 (±0.01)
Instantaneous productivity (g.L^–1^.h^–1^)	0.67 (±0.01)*	0.74 (±0.11)	1.93 (±0.04)
Specific productivity (g.g^–1^)	9.99 (±0.28)	7.13 (±0.65)	15.8 (±0.45)

*In batch mode, the maximum instantaneous productivity of peptides was calculated from 7 to 24 h of culture.

### Spray Drying Optimization and Analysis of the Resulting Ingredient

The continuous process in MBR allowed to produce by fermentation, a semi-purified product containing, among others, peptides of interest and lactic acid with respective concentrations of 10.51 g.L^–1^ and 29.73 g.L^–1^. The pH was equal to 5.95 and dry matter content was 7.86%. Spray drying optimization of the product was conducted based on the maltodextrin concentration required to improve the atomization yield. Maltodextrin concentrations were tested from 0 to 20% (w/v) and the dry matter yields were calculated after spray drying process. Atomization without maltodextrin led to a yield of 0% due to the stickiness of the resulting product which was impossible to collect after drying. A yield of 38% (±7%) of dry matter was obtained for a 5% maltodextrin concentration. Concentrations of 10 and 20% of maltodextrin led to dry matter yields of 66% (±6%) and 72% (±2%), respectively, with no statistical differences reported between these values. Subsequently, a maltodextrin concentration of 10% was selected for the spray drying process of the fermented product.

The fermented product was then tested for its ACE-inhibitory activity before and after atomization. The non-atomized product (P 45a) showed an IC_50_ of 1.87 mg.mL^–1^ (Supplementary Figure S1). A 10% maltodextrin solution was also tested and showed no ACE-inhibitory activity (data not shown). The IC_50_ of the atomized product P 45a A was equal to 1.23 mg.mL^–1^ (corrected to take into account the maltodextrin weight), which was not statistically different from the non-atomized product, suggesting that spray drying did not alter the ACE-inhibitory capacity of the fermented product. Finally, the IC_50_ of the fermented product obtained by continuous fermentation in MBR was compared with previously obtained fermented products (from batch processes followed by an ultrafiltration step). As reported above, IC_50_ of the F45a UFP was equal to 0.47 mg.mL^–1^ and to 8.37 mg.mL^–1^ for the control condition (FCtl UFP). Interestingly, no statistical differences were observed between the previously obtained F45a UFP and the product resulting from continuous fermentation (P 45a A). These results show the robustness and repeatability of biologically active peptide production during fermentation with the *L. helveticus* 45a strain. Moreover, they demonstrate the feasibility of an MBR-based continuous process for the production of a bioactive ingredient aimed at ACE inhibition.

## Conclusion

Batch fermentation leads to the production of fermented milks containing ACE-inhibitory peptides. However, the benefits of fermentation (higher concentration of potentially active peptides compared to the control, increased activity in biochemical assay) are lost when samples are subsequently subjected to SGID. Adding a step of ultrafiltration following fermentation allows to maintain the statistical differences between the fermented products and the non-fermented control, even after SGID. Finally, the feasibility of an MBR-based continuous production process of an active ingredient was demonstrated with an increase in productivity. Further developments are needed to improve the productivity of this process, such as optimization of the dilution rate.

## Data Availability Statement

The raw data supporting the conclusions of this article will be made available by the authors, without undue reservation.

## Author Contributions

CR, BC, and FC conceptualized the experiments. CR, BD, and EB took part in the investigation, methodology, and analysis. CR and FC wrote the manuscript. FC, BC, MF, DD, PD, and CF supervised and helped in the critical review and the editing of the manuscript and participated in the funding acquisition. All authors contributed to the article and approved the submitted version.

## Conflict of Interest

CR and MF are affiliated with VF Bioscience SAS, France, a company commercializing lactic acid bacteria strains for use as probiotics or dairy starters. The remaining authors declare that the research was conducted in the absence of any commercial or financial relationships that could be construed as a potential conflict of interest.
