# Supplementary material for: Integrated Continuous Bioprocess Development for ACE-Inhibitory Peptide Production by Lactobacillus helveticus Strains in Membrane Bioreactor
Source: Front Bioeng Biotechnol. 2020 Sep 25;8:585815. doi: 10.3389/fbioe.2020.585815 (PMC7546403; doi:10.3389/fbioe.2020.585815)
Supplement: Supplementary file 1 [file Data_Sheet_1.PDF]

Figure S1:

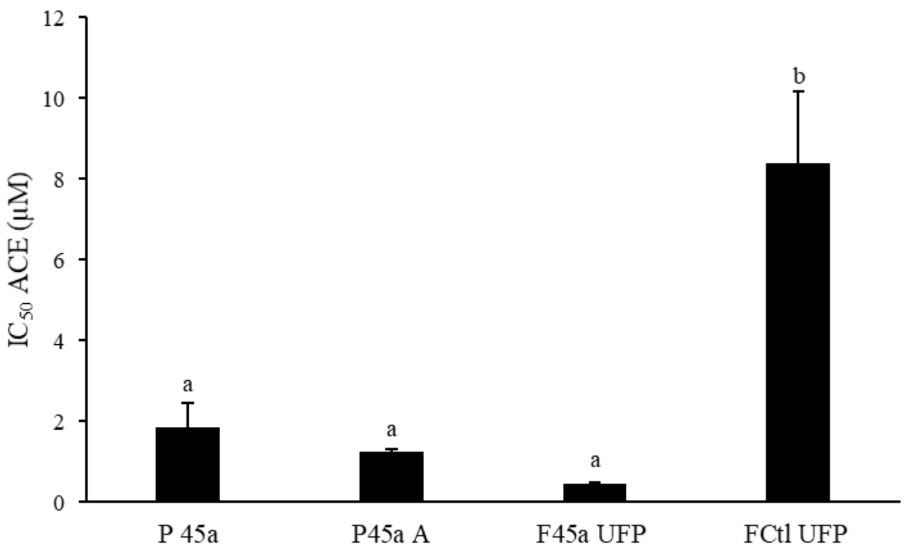

Fig S1. ACE inhibitory IC_{50} values toward of fermented products obtained with the integrated continuous process with the *L. helveticus* 45a strain before and after atomization. IC_{50} were determined in vitro. P45a, non atomized product obtained from continuous milk fermentation in membrane bioreactor with *L. helveticus* 45a strain. P45a A atomized product obtained from continuous milk fermentation in membrane bioreactor with *L. helveticus* 45a strain. F45a UFP, non atomized product obtained from batch fermentation of milk with *L. helveticus* 45a strain followed by an ultrafiltration step. FCtl UFP, non atomized and non fermented milk product submitted to an ultrafiltration step. Data were represented as mean ± SD of three independent experiments. *Statistical analysis was performed using one-way ANOVA and Tukey's post-hoc test. Mean values without a common letter differ significantly (p value < 0.05).

Table S1: Identified peptides from fermented products before and after SGID. For each product, only peptide sequences found in the three replicates of SGID are represented. Each peptides is associated to his parental protein or accession number in Uniprot. m/z: monoisotopic mass/charge ratio; -10logP: peptide identification score; ACE IC_{50} (µM): Predicted IC_{50} from QSAR model toward ACE inhibition. β-LGB : β-lactoglobulin.

Protein	Sequence	m/z	-10logP	ACE IC_{50} (µM)
	F Ctl Raw			
β-casein	GPFPIIV	742,4	33,9	866,0
	KVKEAMAPK	1001,6	49,0	422,2
	MPIQAFLLYQEPVLGPVRGPFPIIV	932,2	44,4	866,0
	PVLGPVRGPFPIIV	731,0	53,0	866,0
	QEPVLGPVRGPFPIIV	859,5	44,4	866,0
	RELEELNVPGEIVESL	913,5	57,9	91,9
	RELEELNVPGEIVESLSSSEESITR	934,8	51,5	373,8
	RELEELNVPGEIVESLSSSEESITRIN	1010,5	40,9	55,6
	SLVYPFPGPIPN	650,8	39,2	18,7
	YQEPVLGPVRGPFPIIV	941,0	74,2	866,0
αs1-casein	APFPEVF	806,4	46,4	87,5
	APFPEVFG	863,4	45,3	1492,1
	APSFSDIPNPIGSEN	772,9	36,2	20,1
	APSFSDIPNPIGSENSEK	944,9	41,3	453,6
	FVAPFPEVFGK	619,3	40,2	91,9
	HIQKEDVPSERYLGYLEQLLRLK	707,6	50,9	1412,2
	IPNPIGSENSEK	642,8	34,5	453,6
	IPNPIGSENSEKTTMPLW	1015,5	46,3	72,5
	IPNPIGSENSEKTTMPLW	1007,5	57,7	72,5
	NPIGSENSEKTTMPLW	902,4	51,0	72,5
	PIGSEN	616,3	34,2	20,1
	PIGSENSEKTTMPLW	853,4	52,9	72,5
	SEKTTMPLW	1092,5	56,7	72,5
	SFSDIPNPIGSENSEK	860,9	37,2	453,6
	VPQLEIVPNSAEERLHSM	683,7	53,4	65,9
G3N0E4	PLGSEN	616,3	34,2	20,1
J9UHS4	SLVYPFPGPIHN	670,9	56,5	20,5
K9ZSD8	EVIESPPEINTVQVTST	922,0	55,9	35,9
	PEVIESPPEINTVQVTSTAV	1055,5	53,7	266,3
	TVQVTSTAV	905,5	49,5	266,3
	VIESPPEINTVQVTSTAV	942,5	44,4	266,3

Protein	Sequence	m/z	-10logP	ACE IC_{50} (µM)
	F 45a Raw			
α-casein	DELQDKIHPF	621,3	53,6	44,6
	DKIHPF	756,4	31,9	44,6
	DMPIQAF	821,4	47,0	40,7
	DVENLHLPLPL	630,4	45,9	160,3
	DVENLHLPLPLLQ	750,9	51,5	111,4
	EDELQDKIHPF	685,8	64,2	44,6
	EEQQQTEDELQDKIHPF	705,3	54,1	44,6
	ELEEL	632,3	28,7	172,3
	EQQQTEDELQDKIHPF	662,3	46,3	44,6
	LLYQEPVLGPVRGPFP	891,5	72,3	109,9
	LLYQEPVLGPVRGPFPIIV	1054,1	54,2	866,0
	LPVPQKAVPYPQ	668,9	31,6	33,3
	LTDVEN	690,3	33,4	20,1
	LYQEPVLGPVRGPFP	835,0	61,6	109,9
	LYQEPVLGPVRGPFPIIV	997,6	55,3	866,0
	QDKIHPF	884,5	34,1	44,6
	QEPVLGPVRGPFP	696,9	47,5	109,9
	QEPVLGPVRGPFPIIV	859,5	42,8	866,0
	QQQTEDELQDKIHPF	619,3	42,5	44,6
	QQTEDELQDKIHPF	864,4	49,0	44,6
	QTEDELQDKIHPF	800,4	51,7	44,6
	SEEQQQTEDELQ	732,3	36,8	111,4
	SEEQQQTEDELQD	789,8	43,1	25,9
	SEEQQQTEDELQDK	853,9	60,0	1540,3
	SEEQQQTEDELQDKIHPF	856,6	78,1	44,6
	TEDELQDKI	545,8	36,9	923,2
	TEDELQDKIHPF	736,4	65,2	44,6
	TESQSLTLT	979,5	34,5	209,7
	TLTDVE	677,3	33,3	241,0
	VLPVPQKAVPYPQ	718,4	44,0	33,3
	YQEPVLGPVRGPFP	778,4	64,8	109,9
	YQEPVLGPVRGPFPIIV	941,0	60,0	866,0
αs1-casein	DIKQM	634,3	29,5	53,5
	DIPNPIGSE	941,5	47,3	70,5
	DQAMEDIKQME	669,3	44,4	308,8
	EEIVPN	700,4	31,5	18,7
	FVAPFPE	806,4	41,6	123,0
	LDAYPS	665,3	28,5	133,0
	LDAYPSG	722,3	36,5	209,0
	QLDAYPS	793,4	35,6	133,0
	SEEIVPN	787,4	39,5	18,7
	SSEEIVPN	874,4	34,7	18,7

Protein	Sequence	m/z	-10logP	ACE IC_{50} (µM)
αs1-casein	STEDQAMEDIK	633,8	37,4	1252,9
	STEDQAMEDIKQM	763,3	45,0	53,5
	STEDQAMEDIKQME	827,8	46,0	308,8
	STEDQAMEDIKQMEAE	927,9	42,4	112,3
	TEDQAMEDIKQM	719,8	42,9	53,5
	TEDQAMEDIKQME	784,3	49,8	308,8
	VAPFPE	659,3	31,9	123,0
	VAPFPEVF	905,5	50,3	87,5
A0A140T8A9	IESPPEIN	898,4	42,5	55,6
E1BG60	ELEEI	632,3	28,7	201,5
E1BPK9	HALPPT	635,3	29,0	62,7
F1MHU9	HAIPPT	635,3	29,0	62,7
G3X7X8	EIEEI	632,3	28,7	201,5
J9UHS4	HNSLPQ	695,3	30,5	33,3
Q0VCZ5	DLKQM	634,3	29,5	53,5
Q58D04	EIEEL	632,3	28,7	172,3

Protein	Sequence	m/z	-10logP	ACE IC_{50} (μM)
	F 49d Raw			
β-casein	DELQDKIHPF	621,3	52,8	44,6
	DKIHPF	756,4	33,9	44,6
	DVENLHLPLPLLQ	750,9	36,5	111,4
	EAMAPK	646,3	35,2	422,2
	ELEEL	632,3	25,7	172,3
	ELNVPGE	757,4	31,5	26,8
	ELNVPGEIVE	1098,6	41,5	241,0
	ELQDKIHPF	563,8	39,2	44,6
	EMPFPKYPVEPFTES	899,4	41,6	143,0
	EMPFPKYPVEPFTESQ	963,5	54,0	19,1
	EPVLGPVRGPFPIIV	795,5	42,7	866,0
	FLLYQEPVLGPVRGPFPIIV	1127,7	52,1	866,0
	FLQPEVM	863,4	44,0	225,1
	FPGPIPN	741,4	35,6	18,7
	FPKYPVEPFTESQ	784,9	52,8	19,1
	HKEMPFPKYPVEPF	776,5	48,6	44,6
	HKEMPFPKYPVEPFTESQ	974,4	46,2	19,1
	HLPLPL	689,4	27,9	160,3
	IPPLTQ	668,4	30,1	32,8
	KAVPYPQ	802,4	45,3	33,3
	KVKEAM	705,4	28,6	104,9
	KVLPVPQ	780,5	25,7	33,3
	LLYQEPVL	974,6	42,2	314,1
	LLYQEPVLGPVRGPFP	891,5	68,3	109,9
	LLYQEPVLGPVRGPFPIIV	1054,1	68,3	866,0
	LQPEVM	716,4	32,0	225,1
	LVYPFPGPIPN	607,3	49,3	18,7
	LVYPFPGPIPNSLPQN	877,0	34,4	8,7
	LYQEPVL	861,5	29,8	314,1
	LYQEPVLGPVRGPFP	835,0	60,0	109,9
	LYQEPVLGPVRGPFPIIV	997,6	51,7	866,0
	MPFPKYPVEPFTE	791,4	34,1	121,3
	MPFPKYPVEPFTESQ	898,9	44,5	19,1
	NIPPLTQ	782,4	48,6	32,8
	NIPPLTQTPV	1079,6	45,4	291,8
	NIPPLTQTPVVVPPF	810,0	38,7	44,6
	NLHLPLPL	916,6	43,6	160,3
	NVPGEIVE	856,4	34,3	241,0
	NVPGEIVESL	1056,6	27,5	91,9
	PFPGPIPN	838,4	37,8	18,7
	PGEIVE	643,3	26,6	241,0
	PGPIPN	594,3	23,8	18,7

Protein	Sequence	m/z	-10logP	ACE IC_{50} (µM)
β-casein	PKYPVEPF	976,5	40,5	44,6
	PKYPVEPFTESQ	711,3	43,0	19,1
	PPLTQ	555,3	24,1	32,8
	QEPVLGPVRGPFPIIV	859,5	51,7	866,0
	SLSQSKVLPVPQKAVPYPQ	689,4	32,5	33,3
	SLTLTD	649,3	25,9	55,0
	SLVYPF	725,4	32,0	44,6
	SLVYPFPGPIPN	650,8	61,1	18,7
	SLVYPFPGPIPNSLPQN	920,5	34,8	8,7
	SVLSLS	605,4	22,8	445,0
	TEDELQDKIHPF	736,4	47,7	44,6
	TESQSL	664,3	28,8	91,9
	TLTDVE	677,3	31,5	241,0
	TLTDVEN	791,4	34,8	20,1
	TLTDVENL	904,5	36,7	33,5
	TPVVVPPFL	968,6	44,2	656,1
	TPVVVPPFLQP	597,3	36,4	12,5
	TPVVVPPFLQPE	661,9	59,4	123,0
	TPVVVPPFLQPEVM	776,9	80,8	225,1
	TPVVVPPFLQPEVM	784,9	73,1	225,1
	TPVVVPPFLQPEVMG	805,4	60,1	915,2
	TPVVVPPFLQPEVMG	813,4	41,9	915,2
	TPVVVPPFLQPEVMGV	855,0	58,0	63,5
	TPVVVPPFLQPEVMGV	863,0	53,6	63,5
	TPVVVPPFLQPEVMGVS	898,5	73,9	260,7
	TPVVVPPFLQPEVMGVS	906,5	64,9	260,7
	TPVVVPPFLQPEVMGVSKVK	717,7	46,2	827,2
	TPVVVPPFLQPEVMGVSKVKE	760,8	45,4	605,8
	TPVVVPPFLQPEVMGVSKVKEA	784,4	48,6	161,9
	TPVVVPPFLQPEVMGVSKVKEAMAP	884,2	27,8	24,5
	TPVVVPPFLQPEVMGVSKVKEAMAPK	695,4	51,3	422,2
	TQTPVVVPPFLQPEVM	891,5	26,5	225,1
	VPGEIVE	742,4	27,8	241,0
	VPPFLQPE	926,5	30,8	123,0
	VVPPFLQPEVM	628,3	33,3	225,1
	VVVPPF	657,4	30,3	44,6
	VVVPPFL	770,5	30,6	656,1
	VVVPPFLQPEVM	677,9	40,1	225,1
	VVVPPFLQPEVMG	706,4	32,0	915,2
	VVVPPFLQPEVMGV	755,9	40,9	63,5
	VVVPPFLQPEVMGVS	807,4	27,6	260,7
	VYPFPGPIPN	917,3	62,7	18,7
	VYPFPGPIPNSLPQN	820,4	29,1	8,7

Protein	Sequence	m/z	-10logP	ACE IC_{50} (μM)
β-casein	YPFPGPIPN	1001,5	48,6	18,7
	YPVEPF	751,4	29,3	44,6
	YQEPVL	748,4	29,8	314,1
	YQEPVLGPVRGPFP	778,4	55,5	109,9
	YQEPVLGPVRGPFPIIV	941,0	62,2	866,0
αs1-casein	DIKQM	634,3	26,3	53,5
	DIPNPIGSE	941,5	38,6	70,5
	DIPNPIGSENSE	848,1	46,5	70,5
	DIPNPIGSENSEK	700,3	59,1	453,6
	DIPNPIGSENSEKTTMP	915,4	28,1	67,4
	EDIKQM	763,4	27,7	53,5
	EMPFPKYPVEPFTE	855,9	51,7	121,3
	EVLNEN	717,3	26,8	20,1
	EVLNENL	830,4	26,6	33,5
	EVLNENLL	943,5	26,5	536,3
	EVLNENLLRF	623,8	28,8	333,8
	EVLNENLLRFF	697,4	37,6	182,7
	FFVAPFPE	953,5	50,7	123,0
	FSDIPNPIGSENSEK	817,4	60,8	453,6
	FSDIPNPIGSENSEKTTMP	1032,5	37,0	67,4
	FVAPFPE	806,4	39,1	123,0
	FVAPFPEVF	1052,5	56,9	87,5
	GKEKVNEL	916,5	31,7	172,3
	GYLEQL	722,4	32,6	74,6
	GYLEQLL	835,5	28,2	536,3
	HQGLPQ	679,4	33,2	33,3
	HQGLPQE	808,4	35,8	57,2
	HQGLPQEVLNENLL	802,4	33,7	536,3
	IGVNQEL	772,4	29,6	172,3
	KHQGLPQ	807,5	39,1	33,3
	KHQGLPQE	936,5	31,5	57,2
	KHQGLPQEVL	574,8	33,7	314,1
	KYKVPQLEIVPN	714,4	28,2	18,7
	LEIVPN	684,4	27,5	18,7
	LLRFFVAPFPE	668,4	33,5	123,0
	LRFFVAPFPE	611,8	29,0	123,0
	RPKHPI	747,5	27,3	187,5
	SDIPNPIGSENSE	679,8	24,5	70,5
	SDIPNPIGSENSEK	743,9	54,1	453,6
	SDIPNPIGSENSEKTTMPLW	1108,5	37,2	72,5
	SFSDIPNPIGSENSEK	860,9	40,5	453,6
	SFSDIPNPIGSENSEKTTMP	1076,0	36,2	67,4
	TDAPSFS	724,3	34,3	544,5

Protein	Sequence	m/z	-10logP	ACE IC_{50} (μM)
αs1-casein	TTMPLW	748,4	25,2	72,5
	VAPFPE	659,3	36,7	123,0
	VAPFPEVF	905,5	48,6	87,5
	VFGKEKVNEL	775,4	35,7	172,3
	YLEQL	665,3	21,3	74,6
	YTDAPS	653,3	34,2	133,0
	YTDAPSFS	887,4	45,8	544,5
αs2-casein	AVPITPT	698,4	25,7	62,7
	AVPITPTLN	925,5	46,0	62,7
	KNTME	622,3	23,7	308,8
	NAVPITPT	812,4	41,5	62,7
A0A0M3R7C5	TIASGEPTSTPTTE	696,3	37,6	121,3
A6QNW3	DAAGGPGAPADPGRPT	703,8	39,2	62,7
AGQNW3	ALLDPS	615,3	23,0	133,0
E1B7K5	YIEQL	665,3	21,3	74,6
E1BC95	LPPITQ	668,4	30,1	32,8
	NLPPITQ	782,4	48,6	32,8
E1BD39	EIEEL	632,3	25,7	172,3
E1BE87	DLKQM	634,3	26,3	53,5
	EDLKQM	763,4	27,7	53,5
E1BEB3	GYLEQI	722,4	32,6	87,2
	YLEQI	665,3	21,3	87,2
E1BGJ9	PPITQ	555,3	24,1	32,8
E7E1Q1	AVESTV	605,3	31,0	287,7
	AVESTVATL	890,5	39,3	158,1
	DKTEIPTIN	1030,5	38,9	55,6
	EIPTIN	686,4	30,3	55,6
	QVTSTAV	705,4	26,8	266,3
	SPPEIN	656,3	37,6	55,6
	STVATLE	720,4	26,8	411,5
	TIASGEPT	775,4	28,8	62,7
	TIASGEPTSTPTIE	702,3	24,4	365,1
	TLEASPE	746,4	31,3	123,0
	TVQVTSTAV	905,5	48,9	266,3
	VIESPPEIN	997,5	49,1	55,6
	VIESPPEINTVQ	663,4	53,4	65,3
	VIESPPEINTVQVT	763,4	32,5	122,8
	VIESPPEINTVQVTSTAV	837,9	28,9	266,3
	VTSTAV	577,3	26,0	266,3
EPYC	HIPLPL	689,4	27,9	160,3
F1MF93	ELEEI	632,3	25,7	201,5
F1N0A6	EIEEI	632,3	25,7	201,5
G3MZC0	ELPTLN	686,4	30,3	62,7

Protein	Sequence	m/z	-10logP	ACE IC_{50} (μM)
GLCM1	ILNKPEDETHLE	719,4	36,6	411,5
J9UHS4	LVYPFPGPIHN	627,3	41,7	20,5
	LVYPFPGPIHNSLPQ	840,0	37,1	33,3
	LVYPFPGPIHNSLPQN	897,0	37,7	8,7
	SLVYPFPGPIH	613,8	28,1	144,4
	SLVYPFPGPIHN	670,9	57,9	20,5
	SLVYPFPGPIHNSLPQ	785,4	52,1	33,3
	SLVYPFPGPIHNSLPQN	940,5	50,3	8,7
	VYPFPGPIHN	760,7	50,6	20,5
	VYPFPGPIHNSLPQ	783,4	40,6	33,3
	YPFPGPIHNSLPQ	733,9	42,4	33,3

	F 60b Raw			
β-casein	APKHKEMPFPKYPVEPF	681,4	50,8	44,6
	DELQDKIHPF	621,3	43,3	44,6
	DELQDKIHPFAQTQ	835,4	46,5	32,8
	DMPIQA	674,3	40,7	70,1
	EPVLGPVRGPFP	632,9	51,4	109,9
	EPVLGPVRGPFPIIV	795,5	42,0	866,0
	FPKYPVEPF	1123,6	57,4	44,6
	LLYQEPVLGPVRGPFPIIV	1054,1	43,5	866,0
	NVPGEIVE	856,4	37,8	241,0
	QEPVLGPVRGPFPIIV	859,5	41,6	866,0
	QQQTEDELQ	1118,5	50,9	111,4
	QSEEQQQTEDELQ	796,3	39,3	111,4
	SEEQQQTEDELQ	732,3	49,4	111,4
	SLTLTDVE	877,5	39,4	241,0
	SVLSLSQS	820,4	33,7	61,9
	TEDELQDKIHPF	736,4	44,8	44,6
	TLTDVEN	791,4	39,8	20,1
	TPVVVPPFLQPEVM	776,9	54,4	225,1
	YPFPGPIPN	1001,5	51,4	18,7
	YQEPVLGPVRGPFPIIV	941,0	52,2	866,0
αs1-casein	DIPNPIGSE	941,5	43,1	70,5
	EDIKQMEAE	1092,5	41,9	112,3
	FSDIPNPIGSENSEKTTMP	1032,5	41,5	67,4
	FVAPFPE	806,4	39,1	123,0
	FVAPFPEVF	1052,6	57,7	87,5
	SDIPNPIGSENSEK	743,9	50,2	453,6

Protein	Sequence	m/z	-10logP	ACE IC_{50} (μM)
αs1-casein	SDIPNPIGSENSEKTTMP	959,0	35,1	67,4
	SFSDIPNPIGSENSEKTTMP	1076,0	40,0	67,4
	VAPFPE	659,3	33,0	123,0
	YTDAPSFSDIPNPIGSENSEK	1134,5	51,5	453,6
A0A0M3R7C5	VATLEDSPE	960,5	44,2	123,0
B2Z898	AVESTVATL	890,5	33,3	158,1
	EIPTINT	787,4	37,7	13,1
	IESPPEIN	898,4	44,9	55,6
	SPPEIN	656,3	35,5	55,6
	TVQVTSTAV	905,5	44,9	266,3
	VQVTSTAV	804,4	38,5	266,3
E7E1P8	VIEGPPEIN	967,5	43,2	55,6
J9UHS4	SLVYPFPGPIHNSLPQ	883,5	51,6	33,3

Protein	Sequence	m/z	-10logP	ACE IC_{50} (µM)
	F Ctl I2			
β-casein	AVPYPQ	674,4	33,8	33,3
	AVPYPQRDMPI	643,8	25,3	187,5
	DELQDKIHPF	621,3	31,8	44,6
	DKIHPF	756,4	27,0	44,6
	DMPIQ	619,3	29,4	98,8
	DMPIQA	690,3	35,1	70,1
	EAMAPK	662,3	31,5	422,2
	EELNVPGE	886,4	33,3	26,8
	EMPFPK	764,4	33,2	422,2
	EMPFPK	748,4	34,5	44,6
	EMPFPKYPVEPF	740,9	32,6	111,4
	EPVLGPV	710,4	28,4	291,8
	FPPQSV	674,4	30,8	167,3
	GPFPII	643,4	26,6	556,4
	GPFPIIV	742,4	34,9	866,0
	GPVRGPFPI	939,5	39,9	187,5
	GPVRGPFPIIV	576,3	31,1	866,0
	HKEMPFPK	858,1	32,3	422,2
	HLPLPL	689,4	26,6	160,3
	HQPHQPLPPT	1151,6	58,7	62,7
	HQPHQPLPPTV	625,8	34,1	287,7
	HQPHQPLPPTVM	691,4	47,6	225,1
	IPPLTQTPVVVPPFLQPEV	921,1	35,9	313,5
	KAVPYPQ	802,4	43,4	33,3
	KIHPF	641,4	32,1	44,6
	LEELNVPGE	999,5	32,9	26,8
	LHLPLP	689,4	23,7	89,8
	LHLPLPL	802,5	28,0	160,3
	LNVPGEI	741,4	30,4	201,5
	LNVPGEIVE	969,5	38,5	241,0
	LQDKIHPF	831,5	38,6	44,6
	LTDVEN	690,3	31,2	20,1
	LTDVENL	803,4	30,5	33,5
	LTDVENLHLPLPL	737,4	57,0	160,3
	LTLTDVEN	904,5	40,5	20,1
	LTLTDVENLHLPLPL	844,5	31,9	160,3
	LVYPFPGPI	1002,6	54,4	187,5
	LVYPFPGPIPNS	650,8	35,4	27,8
	LYQEPVLG	918,5	42,9	1219,6
	LYQEPVLGPV	1114,6	52,8	291,8
	MHQPHQPLPPT	649,8	44,1	422,2

Protein	Sequence	m/z	-10logP	ACE IC_{50} (μM)
β-casein	MHQPHQPLPPT	641,8	60,4	287,7
	MHQPHQPLPPTV	691,4	55,9	225,1
	MHQPHQPLPPTVM	756,9	59,0	225,1
	MHQPHQPLPPTVM	764,9	56,1	225,1
	NIPPLTQT	883,5	44,9	29,2
	NIPPLTQTPVVVPPFLQPEV	1093,1	26,3	313,5
	NLHLPLPL	916,6	37,0	160,3
	NVPGEIVE	856,4	31,6	241,0
	PEVMGVS	734,3	33,9	260,7
	PFPGPIPN	838,4	42,5	18,7
	PFTESQ	708,3	30,6	19,1
	PFTESQS	795,4	35,3	61,9
	PPFLQPEV	926,5	30,6	313,5
	PPFLQPEVMG	1114,6	28,5	915,2
	PPLTQT	656,4	26,6	29,2
	PQNIPPLTQT	1108,6	52,0	29,2
	PQNIPPLTQTPVVVPPF	922,5	48,7	44,6
	PQNIPPLTQTPVVVPPFLQPEV	804,1	68,9	313,5
	PVEPFT	689,4	36,4	256,5
	PVLGPVRGPFPI	624,9	30,3	187,5
	PVVVPPF	754,4	36,2	44,6
	PVVVPPFL	867,5	39,5	656,1
	PVVVPPFLQ	995,6	42,4	111,4
	PVVVPPFLQPE	611,3	46,9	123,0
	PVVVPPFLQPEV	660,9	59,2	313,5
	PVVVPPFLQPEVM	726,4	71,9	225,1
	PVVVPPFLQPEVM	734,4	68,2	225,1
	PVVVPPFLQPEVMG	762,9	71,1	915,2
	PVVVPPFLQPEVMGV	812,4	66,5	915,2
	PVVVPPFLQPEVMGVS	856,0	57,8	63,5
	PVVVPPFLQPEVMG	754,9	67,1	260,7
	PVVVPPFLQPEVMGVS	848,0	40,2	260,7
	QDKIHPF	884,5	33,3	44,6
	QEPVLGPV	838,5	41,6	291,8
	QPLPPTVM	882,5	38,3	225,1
	QSLVYPFPGPIPN	714,9	40,0	18,7
	SLPQNIPPLT	1079,6	34,1	209,7
	SLPQNIPPLTQTPVVVPPFLQPEV	870,8	46,7	313,5
	SLTLTDVEN	991,5	33,0	20,1
	SLTLTDVENL	1104,6	32,7	33,5
	SLVYPFPGPI	1089,6	62,2	187,5
	SLVYPFPGPIPN	650,8	41,1	18,7
	TDVENL	690,3	27,5	33,5

Protein	Sequence	m/z	-10logP	ACE IC_{50} (μM)
β-casein	TDVENLHLPLP	624,3	34,7	89,8
	TDVENLHLPLPL	680,9	60,8	160,3
	TEDELQDKIHP	662,8	36,4	29,4
	TEDELQDKIHPF	736,4	45,4	44,6
	TLTDVEN	791,4	44,0	20,1
	TLTDVENL	904,5	42,6	33,5
	TLTDVENLHLPLPL	787,9	55,5	160,3
	TPVVVPPFLQPEV	711,4	46,8	313,5
	VENLHLPLPL	763,5	34,9	160,3
	VPPFLQPEV	1025,6	34,7	313,5
	VPYPQ	603,3	31,6	33,3
	VVPPFLQPEV	937,4	32,7	313,5
	VYPFPG	679,3	30,1	364,6
	VYPFPGPI	889,5	53,1	187,5
	VYPFPGPIPN	1100,6	66,7	18,7
	WMHQPHQPLPPT	742,9	46,0	48,7
	WMHQPHQPLPPTVM	857,9	45,1	62,7
	WMHQPH	835,4	27,5	62,7
	WMHQPHQPLPPT	734,9	60,9	287,7
	WMHQPHQPLPPTV	784,4	58,2	225,1
	WMHQPHQPLPPTVM	849,9	68,5	225,1
	WMHQPHQPLPPTVM	857,9	44,6	225,1
	WMHQPHQPLPPTVMFPPQSV	790,7	38,2	167,3
	YPFPGPI	790,4	35,9	187,5
	YPFPGPIPN	1001,5	48,1	18,7
	YPVEPF	751,4	33,4	44,6
	YPVEPFT	852,4	31,8	256,5
	YQEPV	635,3	26,7	291,8
	YQEPVLG	805,4	40,7	1219,6
	YQEPVLGPV	1001,5	49,2	291,8
	YQEPVLGPVRGPFPI	835,0	53,9	187,5
	YQEPVLGPVRGPFPIIV	941,0	38,9	866,0
αs1-casein	APFPEV	659,3	32,2	313,5
	APFPEVFG	863,4	42,3	1492,1
	AYFYPEL	902,4	38,8	172,3
	DAYPSGA	680,3	33,9	32,8
	EDVPSE	675,3	24,8	70,5
	EGIHAQQ	782,4	34,0	15,5
	EGIHAQQKEPMIGVN	825,9	42,3	36,7
	EKTTMPL	819,4	29,8	160,3
	EPMIGVN	775,4	38,9	63,5
	EPMIGV	645,3	26,4	36,7
	EPMIGVN	759,4	35,7	36,7

Protein	Sequence	m/z	-10logP	ACE IC_{50} (μM)
αs1-casein	EPMIGVNQ	887,4	34,9	7,0
	FFVAPFPEV	1052,6	51,5	313,5
	FVAPFPEV	905,5	32,0	313,5
	FVAPFPEVFG	1109,6	58,9	1492,1
	HIQKEDVPSE	591,3	43,9	70,5
	HIQKEDVPSER	669,3	52,2	407,3
	HPIKH	631,4	24,6	239,6
	HQGLPQ	679,4	29,2	33,3
	HQGLPQE	808,4	43,3	57,2
	HQGLPQEV	907,5	40,9	313,5
	IGVNQE	659,3	28,8	57,2
	IPNPIGSENSEK	642,8	34,7	453,6
	IQKEDVPSE	1044,5	32,8	70,5
	KHQGLPQ	807,4	34,4	33,3
	KHQGLPQEV	1035,6	36,0	313,5
	LDAYPSGA	793,4	37,0	32,8
	MKEGIHA	785,4	36,7	165,0
	PFPEVFG	792,4	33,7	1492,1
	PMIGVN	630,3	26,2	36,7
	QGLPQEV	770,4	34,3	313,5
	QKEPMIG	802,4	31,1	1081,9
	QKEPMIGV	901,5	32,3	63,5
	QKEPMIGVN	1015,5	42,1	36,7
	QKEPMIGVNQEL	693,4	34,6	172,3
	QLDAYPS	793,4	36,4	133,0
	QLDAYPSGA	921,4	46,3	32,8
	QQKEPMIGV	1029,5	36,6	63,5
	SDIPNPI	755,4	27,0	187,5
	SDIPNPIG	812,4	30,3	1081,9
	SDIPNPIGSE	1028,5	40,5	70,5
	SDIPNPIGSEN	1142,5	58,9	20,1
	SDIPNPIGSENSEK	743,9	67,0	453,6
	SDIPNPIGSENSEKT	794,4	45,4	308,6
	SFSDIPNPI	989,5	46,2	187,5
	VAPFPEV	758,4	40,6	313,5
	VAPFPEVF	905,5	37,3	87,5
	VAPFPEVFG	962,5	36,4	1492,1
	VAPFPEVFGKE	1016,5	49,9	605,8
	VPLGTQ	614,4	33,8	32,8
	YFYPEL	831,4	32,7	172,3
	YLGYLEQLLR	634,4	33,1	1267,9
	YQLDAYPS	956,4	31,5	133,0
	YQLDAYPSGA	1084,5	48,8	32,8

Protein	Sequence	m/z	-10logP	ACE IC_{50} (μM)
αs1-casein	YTDAPS	653,3	32,4	133,0
	YTDAPSF	800,3	37,5	25,6
	YTDAPSFSDIPNPI	768,9	32,1	187,5
	YVPLGT	649,4	24,9	13,6
	YVPLGTQ	777,4	41,3	32,8
αs2-casein	ALNEINQ	801,4	28,0	7,0
	AVPITPT	698,4	27,7	62,7
	ITVDDK	690,4	28,4	1540,3
	ITVDDKH	827,4	40,9	239,6
	LYQGPIV	789,5	49,4	866,0
	NAVPIT	614,4	25,0	186,0
	NAVPITPT	812,4	45,0	62,7
	NPWDQV	758,3	36,0	135,8
	PIVLNPWDQV	787,4	39,4	135,8
	QGPIVLNPWDQV	683,4	34,6	135,8
	TVDMESTEV	1010,4	32,4	313,5
	VKITVDDK	917,5	41,7	1540,3
	VLNPWDQV	970,5	43,1	135,8
	YQGPIV	676,4	31,5	866,0
	YQGPIVLNPWDQ	715,4	39,5	121,5
	YQGPIVLNPWDQV	764,9	45,9	135,8
	YQGPIVLNPWDQVK	828,9	48,2	827,2
	YQKFPQ	810,4	33,1	33,3
β-LGB	EKFDK	666,3	31,4	1540,3
	ELKPTPEGDL	1098,6	44,2	584,9
	IDALNE	674,3	30,0	25,7
	IDALNEN	788,4	31,3	20,1
	ISLLDAQSAPL	1127,6	33,1	160,3
	IVTQTM	692,4	32,9	113,3
	KIDALNE	802,4	41,5	25,7
	KPTPEGDL	856,4	35,8	584,9
	LIVTQT	674,4	31,9	29,2
	LIVTQTM	805,4	45,5	113,3
	LIVTQTMK	933,5	48,8	1059,7
	LKPTPEGDL	969,5	42,3	584,9
	LVLDTDY	838,4	36,2	685,2
	SDISLLDAQSAPLRV	792,9	32,3	2181,7
	SLAMAASDISL	730,0	29,7	684,1
	SLAMAASDI	878,4	45,5	394,8
	SLAMAASDIS	965,5	42,3	91,9
	SLAMAASDISL	1078,5	44,3	91,9
	SLAMAASDISLLDAQSAPL	937,5	64,2	160,3
	SLAMAASDISLLDAQSAPLR	1015,5	82,6	1267,9

Protein	Sequence	m/z	-10logP	ACE IC_{50} (μM)
β-LGB	SLAMAASDISLLDAQSAPLRV	946,8	54,6	2181,7
	SLLDAQ	646,3	27,5	30,4
	SLLDAQSAPL	1014,5	33,1	160,3
	TPEVDDEAL	988,4	44,3	146,3
	TPEVDDEALEK	623,3	49,4	453,6
	TPEVDDEALEKFDK	727,6	55,9	1540,3
	VEELKPTPEGDL	663,8	63,9	584,9
	VEELKPTPEGDLE	728,4	52,5	411,5
	VEELKPTPEGDLEI	784,9	53,4	201,5
	VEELKPTPEGDLEIL	841,4	53,7	475,8
	VLDTDY	725,3	35,1	685,2
	VLDTDYK	853,4	43,4	828,7
	VLVLDTD	774,4	31,3	55,0
	VLVLDTDY	937,5	48,3	685,2
	VLVLDTDYK	1065,6	41,1	828,7
	VRTPEVDD	930,5	36,6	203,3
	VRTPEVDDEA	754,0	47,5	161,9
	VYVEELKPTPEGD	738,4	36,8	12,1
	VYVEELKPTPEGDL	794,9	48,1	584,9
	VYVEELKPTPEGDLEI	916,0	50,4	201,5
	VYVEELKPTPEGDLEIL	972,5	62,7	475,8
	YVEELKPTPEGD	688,8	53,9	12,1
	YVEELKPTPEGDL	745,4	64,6	584,9
	YVEELKPTPEGDLE	809,9	54,3	411,5
	YVEELKPTPEGDLEI	866,4	46,3	201,5
	YVEELKPTPEGDLEIL	923,0	42,7	475,8
Pepsin	AIVDTGTS	763,4	41,8	131,2
	AIVDTGTSL	876,5	45,5	91,9
	DTVQVGGI	788,4	36,9	40,8
	FDNIWDQG	994,4	43,8	169,6
	FDNIWDQGLV	804,7	45,3	976,1
	FDTGSSNL	840,4	38,8	33,5
	FGTIGIGTPA	933,5	35,3	150,7
	GGISDTNQI	904,4	31,0	87,2
	GILGYDTV	837,4	46,4	287,7
	GLAYPSIS	807,4	30,5	394,8
	GLSETEPGS	876,4	33,9	29,0
	GTIGIGTPA	786,4	36,7	150,7
	GTPAQDF	735,3	34,8	162,9
	GTPAQDFT	836,4	35,1	256,5
	IGTPAQD	701,3	34,1	25,9
	SIDSLPDI	859,4	29,8	684,1
	SIDSLPDIV	958,5	43,1	866,0

Protein	Sequence	m/z	-10logP	ACE IC_{50} (μM)
Pepsin	TVIFDTGSS	926,4	43,9	76,3
	TVIFDTGSSN	1040,5	51,1	10,7
	TVIFDTGSSNL	1153,6	49,0	33,5
	VDTGTSL	692,3	25,3	91,9
A0A0M3R7C5	ARHPHPH	851,4	29,1	48,7
	AVESTV	605,3	30,1	287,7
	EDSPEVIESPPEINT	828,4	39,3	13,1
	ESPPEIN	785,4	34,0	55,6
	ESPPEINT	886,4	47,6	13,1
	FLPYPY	799,4	26,7	187,8
	HPHPH	624,3	29,5	48,7
	IESPPEINT	999,5	43,7	13,1
	LPYPY	652,3	25,3	187,8
	MAIPPK	656,4	27,3	62,7
	NNQFLPYP	992,5	31,3	52,7
	NQDKTEIPTINT	687,3	40,7	13,1
	SPAQILQ	756,4	33,7	111,4
	TEIPTINT	888,5	47,0	13,1
	VIESPPEINT	1098,6	45,1	13,1
	VQVTSTAV	804,4	39,1	266,3
	YAKPAA	620,3	31,6	137,5
	YPSYGLN	813,4	41,7	62,7
	YQQKPVA	833,5	30,3	295,3
A5PJW4	ALVDTGTS	763,4	41,8	131,2
B5B3R8	SDIPNPIGSENSGK	707,8	48,1	91,9
CHIA	SGAGPAGPY	776,4	45,5	187,8
E1AXU0	GPFPLL	643,4	26,6	536,3
E1B9F5	LDAINE	674,3	30,0	25,7
E1BB93	NAVPLT	614,4	25,0	209,7
E1BK38	EMPLQ	633,3	26,9	422,2
E1BMU3	VIVLDTD	774,4	31,3	55,0
E7E1Q1	IASGEPTSTPTIE	651,8	29,1	365,1
EPYC	DNNLDHIPLPL	630,8	35,9	160,3
	HIPLPL	689,4	26,6	160,3
	TDNNLDHIPLPL	681,4	46,4	160,3
F1MIX6	EHVIFPGA	869,4	26,9	32,8
G3N0E0	ALVDTGTSL	876,5	45,5	91,9
J9UHS4	HNSLPQ	695,3	30,2	33,3
	HNSLPQNIPPLTQT	780,4	49,7	29,2
	LVYPFPGPIHN	627,3	34,2	20,5
	LVYPFPGPIHNSLPQ	840,0	42,4	33,3
	QSLVYPFPGPIHN	734,9	40,6	20,5
	QSLVYPFPGPIHNSLPQ	947,5	33,5	33,3

Protein	Sequence	m/z	-10logP	ACE IC_{50} (µM)
J9UHS4	VYPFPGPIHN	1140,6	61,1	20,5
	VYPFPGPIHNS	614,3	36,0	27,8
	VYPFPGPIHNSLPQ	783,4	57,5	33,3
P02663	PIVLNPWD	953,5	45,2	363,2
PIGR	QVLEPEPEL	1053,5	31,9	172,3
Q1RMN8	ISDFYPGSVT	1085,5	33,4	122,8
	SVSGSLGQ	734,4	28,1	7,2
Q28049	DDDLTDDIM	1052,4	28,2	341,0
	DLTDDIM	822,4	28,5	341,0
	FLDDDLTDD	1068,4	33,8	203,3
	GGVSLPEWV	943,5	40,8	1902,0
	GYGGVSLPEWV	1163,6	45,0	1902,0
	ILDKVGIN	871,5	33,2	55,6
	LDDDLTDDIM	1165,5	36,7	341,0
Q28157	DSITMDGETIA	1152,5	31,1	447,2
Q85PG9	TVLFDTGSS	926,4	43,9	76,3
Q95M17	AIDLDDFTGT	1067,5	32,8	13,6
SPT22	NPELPPLTKT	1109,6	34,8	308,6

	F 45a I2			
β-casein	EAMAPK	646,3	37,9	422,2
	EMPFPK	748,4	33,3	422,2
	FPPQSV	674,4	34,8	167,3
	KIHPF	641,4	30,8	44,6
	KVLPVPQ	780,5	26,9	33,3
	LHLPLPL	802,5	27,7	160,3
	LQDKIHPF	997,5	34,3	44,6
	LVYPFPGPI	1002,6	56,2	187,5
	MHQPHQPLPPT	641,8	39,8	62,7
	NIPPLTQT	883,5	44,5	29,2
	NLHLPLPL	916,6	40,2	160,3
	NVPGEI	628,3	26,1	201,5
	PFTESQ	708,3	30,9	19,1
	PFTESQS	795,4	35,0	61,9
	PQNIPPLTQT	1108,6	57,6	29,2
	PVEPFT	689,3	34,8	256,5
	PVVVPPF	754,4	44,4	44,6
	PVVVPPFL	867,5	33,2	656,1
	PVVVPPFLQPEV	660,9	54,3	313,5

Protein	Sequence	m/z	-10logP	ACE IC_{50} (μM)
β-casein	PVVVPPFLQPEVM	726,4	55,0	225,1
	PVVVPPFLQPEVMG	762,9	54,2	915,2
	PVVVPPFLQPEVMG	754,9	55,7	915,2
	SLVYPFPGPI	1089,6	63,8	187,5
	TEDELQDKIHP	662,8	31,0	29,4
	TEDELQDKIHPF	736,4	45,1	44,6
	TQTPVVVPPF	1084,6	35,0	44,6
	VYPFPGPI	889,5	55,3	187,5
	VYPFPGPIPN	917,3	45,8	18,7
	WMHQPHQPLPPT	734,9	52,7	62,7
	YPVEPF	751,4	30,4	44,6
	YQEPV	635,3	28,8	291,8
	YQEPVLG	805,4	38,2	1219,6
αs1-casein	HIQKEDVPSE	984,8	37,8	70,5
	HQGLPQE	808,4	31,3	57,2
	LDAYPSG	722,3	26,7	209,0
	SDIPNPIG	812,4	29,1	1081,9
	VAPFPEV	758,4	29,9	313,5
	VAPFPEVFG	962,5	39,3	1492,1
	YQLDAYPSGA	1084,5	35,6	32,8
	YTDAPS	653,3	28,8	133,0
αs2-casein	ITVDDK	690,4	28,5	1540,3
	ITVDDKH	827,4	29,4	239,6
	NAVPITPT	812,5	44,3	62,7
	VKITVDDK	917,5	32,8	1540,3
	YQGPIV	676,4	28,6	866,0
α-lactalbumin	DDDLTDDIM	1052,4	45,2	341,0
	DLTDDIM	822,4	32,4	341,0
	FLDDDLTD	953,4	34,6	55,0
	GGVSLPEWV	943,5	38,5	1902,0
	IVQNNDSTE	1019,5	43,9	121,3
	LDDDLTDD	921,4	45,8	203,3
	LDDDLTDDI	1034,5	39,5	684,1
	LDDDLTDDIM	1165,5	51,6	341,0
β-LGB	EELKPTPEGDL	614,3	51,9	584,9
	EKFDK	666,4	31,5	1540,3
	EKFDKA	737,4	31,7	742,0
	ELKPTPE	813,4	26,8	123,0
	ELKPTPEGD	985,5	41,3	12,1
	ELKPTPEGDL	915,6	43,6	584,9
	ELKPTPEGDLE	818,7	47,1	411,5
	ELKPTPEGDLEI	670,9	37,3	201,5
	GLDIQK	673,4	28,0	196,4

Protein	Sequence	m/z	-10logP	ACE IC_{50} (μM)
β-LGB	GLDIQKVAGTWY	675,9	42,1	1224,1
	GLDIQKVAGTWYSLAMAASD	1049,0	35,0	32,0
	GLDIQKVAGTWYSLAMAASDI	1105,6	44,7	684,1
	IDALNE	674,3	30,1	25,7
	IVTQTM	692,4	30,9	113,3
	KIDALNE	802,4	42,3	25,7
	KIIAE	573,4	26,8	112,3
	KPTPEGDL	856,4	35,7	584,9
	LIVTQTM	805,4	46,9	113,3
	LIVTQTM	821,4	42,3	113,3
	LIVTQTMK	933,5	53,9	1059,7
	LIVTQTMKGLDIQK	794,5	48,9	196,4
	LKPTPEGD	856,4	34,8	12,1
	LKPTPEGDL	969,5	40,0	584,9
	LKPTPEGDLE	1098,6	34,9	411,5
	LVLDTD	675,4	27,1	55,0
	LVLDTDY	838,4	37,9	685,2
	SLAMAASD	765,3	41,5	32,0
	SLAMAASDI	878,4	52,4	684,1
	SLAMAASDIS	965,5	35,3	394,8
	SLAMAASDISL	1078,5	43,5	91,9
	SLAMAASDISLLDAQ	753,4	37,1	30,4
	SLAMAASDISLLDAQSAPL	937,5	42,9	160,3
	SLLDAQ	646,3	27,2	30,4
	TPEVDD	675,3	35,7	203,3
	TPEVDDE	804,3	40,2	448,8
	TPEVDDEA	875,4	52,3	161,9
	TPEVDDEAL	988,4	50,3	146,3
	VAGTWYSLAMAASDISL	878,4	40,0	91,9
	VAGTWYSLAMAASDISLL	935,0	44,7	536,3
	VEELKPTPE	1041,5	36,8	123,0
	VEELKPTPEGDL	663,8	47,5	584,9
	VEELKPTPEGDLE	728,4	50,3	411,5
	VEELKPTPEGDLEIL	841,4	43,3	475,8
	VLDTD	562,3	24,6	55,0
	VLDTDY	725,3	36,6	685,2
	VLDTDYK	853,4	38,4	828,7
	VRTPEVDD	775,5	40,6	203,3
	VRTPEVDDE	706,7	38,4	448,8
	VRTPEVDDEA	565,8	50,3	161,9
	VTQTMK	707,4	28,2	1059,7
	VYVEELKPTPEGDL	794,9	40,7	584,9
	VYVEELKPTPEGDLEI	916,0	43,1	201,5

Protein	Sequence	m/z	-10logP	ACE IC_{50} (μM)
β-LGB	YVEELKPTPEGD	688,8	39,7	12,1
	YVEELKPTPEGDL	745,4	45,8	584,9
	YVEELKPTPEGDLE	809,9	47,1	411,5
	YVEELKPTPEGDLEIL	923,0	45,7	475,8
Pepsin	AIVDTGT	676,4	36,6	13,6
	AIVDTGTS	763,4	43,4	131,2
	AIVDTGTSL	876,5	46,9	91,9
	DTGSSNL	693,3	34,7	33,5
	DTVQVGGI	788,4	34,6	40,8
	FDNIWDQG	994,4	43,3	169,6
	FDTGSS	613,2	28,3	76,3
	FDTGSSN	727,3	43,9	10,7
	FGTIGIGTPA	933,5	35,6	150,7
	GGISDTN	663,3	36,2	18,5
	GGISDTNQ	791,4	37,7	7,0
	GLAYPSIS	807,4	36,0	394,8
	GTIGIGTPA	786,4	42,7	150,7
	GTIGIGTPAQD	1029,5	57,1	25,9
	GTPAQDF	735,3	42,6	162,9
	IGTPAQD	701,3	38,5	25,9
	IVDTGTS	692,3	37,0	131,2
	IVDTGTSL	805,4	36,9	91,9
	NIWDQGLV	944,5	38,7	976,1
	QVGGISDTN	890,4	52,1	18,5
	QVGGISDTNQ	1018,5	57,6	7,0
	QVGGISDTNQI	1131,6	43,6	87,2
	SIDSLPDIV	958,5	48,2	866,0
	VGGISDT	648,3	30,1	228,7
	VGGISDTNQ	890,4	47,5	7,0
	VIFDTGSS	825,4	44,9	76,3
	WDQGLVS	804,4	26,0	260,7
	WILGDV	702,4	29,6	1064,6
	YAPFDGIL	895,5	26,0	475,8
	YAPFDGILG	952,5	29,8	1219,6
	YGTGSM	615,2	32,0	65,9
A0A0M3R7C5	EIPTINT	787,4	31,6	13,1
	ESPPEIN	785,4	33,5	55,6
	ESPPEINT	886,4	47,5	13,1
	IESPPEIN	898,5	36,3	55,6
	IESPPEINT	999,5	49,0	13,1
	LEDSPEV	788,4	29,6	313,5
	NQDKTEIPT	1045,5	27,7	62,7
	TEIPTIN	787,4	34,1	55,6

Protein	Sequence	m/z	-10logP	ACE IC_{50} (µM)
A0A0M3R7C5	TEIPTINT	888,5	47,1	13,1
	VQVTSTAV	804,4	42,6	266,3
	YAKPAA	620,3	26,3	137,5
A5PJW4	ALVDTGT	676,4	36,6	13,6
	ALVDTGTS	763,4	43,4	131,2
	LVDTGTS	692,3	37,0	131,2
A5PJW4	WLLGDV	702,4	29,6	1064,6
CATD	YTVFD	644,3	26,5	228,1
E1BKJ9	KILAE	573,4	26,8	112,3
E7E1P8	IEGPPEINT	969,5	37,7	13,1
F1MH27	AIDLDDFTGT	1067,5	33,9	13,6
	SGAGPAGPY	776,4	36,1	187,8
F1MLW7	SVSGSLGQ	734,4	31,6	7,2
	YQQVPGSGL	948,5	36,4	34,9
F1MP21	ALVFVDNHDNQR	714,4	50,6	176,4
	NVVDGQPF	875,4	36,8	44,6
	SISNSAEDPF	1066,5	43,6	44,6
F1MP81	LDALNE	674,3	30,1	25,7
F1MPA5	DTGSSNI	693,3	34,7	39,2
F1MWS3	VLFDTGSS	825,4	44,9	76,3
F1N0T6	KLIAE	573,4	26,8	112,3
G3N0E0	LVDTGTSL	805,4	36,9	91,9
	ALVDTGTSL	876,5	46,9	91,9
	SYMPEPVT	923,4	42,9	122,8
	YMPEPVT	836,4	30,3	122,8
G5E533	LDAINE	674,3	30,1	25,7
KASH5	KLLAE	573,4	26,8	112,3
Q28157	DSITMDGET	968,4	41,8	67,3
	DSITMDGETI	1081,5	38,6	184,9
	YTGSLN	654,3	30,2	62,7
Q3MHH8	NWGEGWGFMPSDR	769,8	40,4	1382,9
Q6LBN7	FGSPPGQ	689,3	29,9	7,2
	VTLDGGM	692,3	34,7	25,0

Protein	Sequence	m/z	-10logP	ACE IC_{50} (µM)
	F 49d I2			
β-casein	AVPYPQ	674,4	30,0	33,3
	AVPYPQRDMPIQ	707,9	33,7	98,8
	DKIHPF	756,4	25,9	44,6
	DMPIQ	619,3	28,5	98,8
	DMPIQA	690,3	34,3	98,8
	DMPIQ	603,3	25,1	70,1
	EAMAPK	662,3	33,8	422,2
	EELNVPGE	886,4	34,5	26,8
	ELNVPGE	757,4	29,5	26,8
	EMPFPK	764,4	34,1	422,2
	EPVLGPV	710,4	29,3	291,8
	FPPQSV	674,4	33,6	167,3
	GPFPII	643,4	24,6	556,4
	GPFPIIV	742,4	32,0	866,0
	GPVRGPFPI	626,7	41,2	187,5
	HKEMPFPK	686,7	41,3	422,2
	HLPLPL	689,4	24,5	160,3
	HQPHQPLPPT	1151,6	59,5	62,7
	HQPHQPLPPTV	625,8	32,9	287,7
	HQPHQPLPPTVM	691,4	46,3	225,1
	IPPLTQT	769,4	31,0	29,2
	KAVPYPQ	802,4	39,7	33,3
	KIHPF	641,4	30,3	44,6
	LHLPLPL	802,5	28,5	160,3
	LNVPGEI	741,4	26,6	201,5
	LNVPGEIVE	969,5	34,9	241,0
	LTDVEN	690,3	29,0	20,1
	LTDVENLHLPLPL	737,4	29,3	160,3
	LTLTDVEN	904,5	35,3	20,1
	LVYPFPGPI	1002,6	50,3	187,5
	LYQEPV	748,4	28,1	291,8
	LYQEPVLG	918,5	42,1	1219,6
	MHQPHQPLPPT	641,8	61,6	62,7
	MHQPHQPLPPTV	691,4	48,6	287,7
	MHQPHQPLPPTVM	756,9	56,1	225,1
	NIPPLTQT	883,5	43,4	29,2
	NIPPLTQTPVVVPPFLQPEV	1093,1	35,8	313,5
	NLHLPLP	803,5	26,4	89,8
	NLHLPLPL	916,6	38,8	160,3
	NVPGEIVE	856,4	33,5	241,0

Protein	Sequence	m/z	-10logP	ACE IC_{50} (µM)
β-casein	PEVMGV	647,3	34,5	63,5
	PFPGPIPN	838,4	42,0	18,7
	PFTESQ	708,3	31,0	19,1
	PFTESQS	795,3	36,5	61,9
	PGPIPN	594,3	22,9	18,7
	PPFLQPEV	926,5	31,4	313,5
	PPLTQT	656,4	26,8	29,2
	PQNIPPLTQT	924,0	58,0	29,2
	PQNIPPLTQTPVVVPPFLQPEV	804,1	42,3	313,5
	PVEPF	588,3	23,0	44,6
	PVEPFT	689,4	36,2	256,5
	PVVVPPF	754,4	41,8	44,6
	PVVVPPFL	867,5	38,9	656,1
	PVVVPPFLQPEV	660,9	56,2	313,5
	PVVVPPFLQPEVM	726,4	74,1	225,1
	PVVVPPFLQPEVM	734,4	70,1	225,1
	PVVVPPFLQPEVMG	762,9	68,2	915,2
	PVVVPPFLQPEVMGV	812,4	62,8	915,2
	PVVVPPFLQPEVMGVS	856,0	64,7	63,5
	PVVVPPFLQPEVMG	754,9	73,2	260,7
	PVVVPPFLQPEVMGVS	848,0	42,3	260,7
	QDKIHPF	884,5	28,3	44,6
	QEPVLGPV	838,5	36,3	291,8
	QPLPPTVM	882,5	34,8	225,1
	SLPQNIPPLT	1079,6	30,2	209,7
	SLPQNIPPLTQT	654,9	45,5	29,2
	SLVYPFPGPI	1089,6	71,3	187,5
	TDVENL	690,3	28,8	33,5
	TDVENLHLPLPL	680,9	53,9	160,3
	TEDELQDKIHPF	736,4	37,3	44,6
	TLTDVE	677,3	24,0	241,0
	TLTDVEN	791,4	46,7	20,1
	TLTDVENL	904,5	44,3	33,5
	TPVVVPPF	855,5	36,5	44,6
	TPVVVPPFLQPEV	711,4	54,7	313,5
	TQTPVVVPPFLQPEV	826,0	38,1	313,5
	VENLHLPLPL	1144,7	37,3	160,3
	VYPFPG	679,3	31,6	364,6
	VYPFPGPI	889,5	53,0	187,5
	VYPFPGPIPN	1100,6	60,3	18,7
	VYPFPGPIPNS	989,8	64,5	27,8
	WMHQPHQPLPPT	742,9	42,2	48,7
	WMHQPH	835,4	26,6	62,7

Protein	Sequence	m/z	-10logP	ACE IC_{50} (μM)
β-casein	WMHQPHQPLPPT	734,9	70,0	62,7
	WMHQPHQPLPPTV	784,4	47,2	287,7
	WMHQPHQPLPPTVM	849,9	65,1	225,1
	YPFPGPI	790,4	33,6	187,5
	YPFPGPIPN	1001,5	42,9	18,7
	YPVEPF	751,4	31,3	44,6
	YPVEPFT	852,4	34,4	256,5
	YQEPV	635,3	24,4	291,8
	YQEPVLG	805,4	42,5	1219,6
	YQEPVLGPV	1001,5	43,0	291,8
	YQEPVLGPVRGPF	729,9	37,4	44,6
	YQEPVLGPVRGPFPI	835,0	49,5	187,5
αs1-casein	APFPEV	659,3	28,7	313,5
	AYFYPE	789,3	24,2	123,0
	AYFYPEL	902,4	38,1	172,3
	DAYPSGA	680,3	35,7	32,8
	EKTTMPL	819,4	24,5	160,3
	EPMIGVN	775,4	33,3	36,7
	EPMIGVN	759,4	40,2	36,7
	FSDIPNPI	902,5	30,0	187,5
	FVAPFPEV	905,5	31,3	313,5
	FVAPFPEVFG	924,8	42,0	1492,1
	FYPEL	668,3	23,8	172,3
	HPIKH	631,4	24,2	239,6
	HQGLPQ	679,4	28,0	33,3
	HQGLPQE	808,4	34,4	57,2
	HQGLPQEV	907,5	42,8	313,5
	KTTMPL	690,4	33,2	160,3
	LDAYPS	665,3	23,0	133,0
	LEIVPN	684,4	24,6	18,7
	MKEGIHA	785,4	32,7	165,0
	PFPEVFG	792,4	31,6	1492,1
	PMIGVN	630,3	25,6	36,7
	QGLPQEV	770,4	34,1	313,5
	QKEPMIGV	901,5	35,4	63,5
	QKEPMIGVN	846,4	34,9	36,7
	QLDAYPSGA	921,4	31,9	32,8
	QQKEPMIGV	1029,5	30,1	63,5
	QQKEPMIGVN	1143,6	40,6	36,7
	SDIPNPI	755,4	26,0	187,5
	SDIPNPIG	812,4	34,7	1081,9
	SDIPNPIGSEN	1142,5	55,2	20,1
	SDIPNPIGSENSEK	743,9	68,8	453,6

Protein	Sequence	m/z	-10logP	ACE IC_{50} (μM)
αs1-casein	SDIPNPIGSENSEKT	794,4	34,7	308,6
	SFSDIPNPI	989,5	43,2	187,5
	VAPFPEV	758,4	38,2	313,5
	VAPFPEVFG	962,5	56,0	1492,1
	VPLGTQ	614,4	34,0	32,8
	YFYPEL	831,4	30,8	172,3
	YQLDAYPS	956,4	32,2	133,0
	YQLDAYPSGA	1084,5	50,4	32,8
	YTDAPSF	800,3	39,4	25,6
αs2-casein	AINPSKENL	985,5	31,0	33,5
	AVPITPT	698,4	26,0	62,7
	ITVDDKH	827,4	33,5	239,6
	LYQGPIV	789,5	39,1	866,0
	NAVPITPT	812,5	44,8	62,7
	NPWDQV	758,3	31,4	135,8
	VKITVDDK	917,5	42,0	1540,3
	VLNPWDQV	970,5	38,2	135,8
	YQGPIVLN	903,5	34,5	62,7
	YQKFPQ	810,4	29,4	33,3
K-casein	ARHPHPH	851,5	31,2	48,7
	EIPTINT	787,4	27,8	13,1
	EQNQEQPI	985,5	38,0	187,5
	ESPPEIN	785,4	37,5	55,6
	ESPPEINT	886,4	49,3	13,1
	ESPPEINTV	985,5	40,1	287,7
	IASGEPTSTPT	1060,5	28,5	62,7
	IASGEPTSTPTTE	645,8	45,5	121,3
	IESPPEIN	898,5	41,2	55,6
	IESPPEINT	999,5	46,1	13,1
	INNQFLPYP	921,5	24,4	52,7
	MAIPPK	656,4	27,0	422,2
	NQDKTEIPTINT	687,3	35,7	13,1
	QFLPYPY	927,5	29,2	187,8
	SPAQIL	628,4	25,7	475,8
	SPAQILQ	756,4	33,9	111,4
	SRYPSYG	829,4	31,0	715,6
	TEIPTIN	787,4	32,2	55,6
	TEIPTINT	888,5	45,8	13,1
	VQVTSTAV	804,4	39,3	266,3
	YAKPAA	620,3	29,3	137,5
	YPSYGLN	813,4	31,2	62,7
	YQQKPVA	833,4	30,8	295,3
	YYQQKPVA	996,5	35,5	295,3

Protein	Sequence	m/z	-10logP	ACE IC_{50} (µM)
α-lactalbumin	DDDLTDDIM	1052,4	39,4	341,0
	DLTDDIM	822,4	26,7	341,0
	FLDDDLTDD	1068,4	41,5	203,3
	GGVSLPEW	844,4	30,6	23,3
	GGVSLPEWV	943,5	31,5	1902,0
	ILDKVGIN	871,5	33,9	55,6
	LDDDLTD	806,3	28,9	55,0
	LDDDLTDD	921,4	33,9	203,3
	LDDDLTDDI	1034,5	39,0	684,1
	LDDDLTDDIM	1165,5	43,3	341,0
	YGGVSLPEWV	1106,6	31,3	1902,0
β-LGB	EELKPTPEGDL	614,3	52,1	584,9
	EKFDK	666,3	25,7	1540,3
	ELKPTPEGD	985,5	35,9	12,1
	ELKPTPEGDL	1098,6	47,4	584,9
	GLDIQKVAGTW	594,3	39,4	21,4
	GLDIQKVAGTWY	675,9	46,5	1224,1
	ISLLDAQSAPL	1127,6	27,0	160,3
	IVTQTM	692,4	30,7	113,3
	KIDALNE	802,4	32,3	25,7
	KPTPEGDL	856,4	34,0	584,9
	LIVTQT	674,4	34,2	29,2
	LIVTQTM	805,4	46,5	113,3
	LIVTQTMK	933,5	46,9	1059,7
	LIVTQTMKGLDIQK	794,5	52,2	196,4
	LKPTPEGD	856,4	28,0	12,1
	LKPTPEGDL	969,5	32,7	584,9
	LKPTPEGDLE	732,7	31,5	411,5
	LVLDTDY	838,4	42,3	685,2
	MAASDI	607,3	23,7	684,1
	PTPEGDL	728,3	28,3	584,9
	SFNPTQ	693,3	29,1	32,8
	SLAMAASDI	878,4	39,6	684,1
	SLAMAASDIS	965,5	47,5	394,8
	SLAMAASDISL	1078,5	44,1	91,9
	SLAMAASDISLLDAQ	753,4	43,6	30,4
	SLAMAASDISLLDAQSAPL	937,5	37,3	160,3
	SLAMAASDISLLDAQSAPLRV	1065,1	62,8	2181,7
	SLLDAQSAPL	1014,5	36,5	160,3
	TPEVDDEA	875,4	50,6	161,9
	TPEVDDEAL	988,4	47,6	146,3
	TPEVDDEALEK	623,3	58,5	453,6
	TPEVDDEALEKFDK	818,4	53,8	1540,3

Protein	Sequence	m/z	-10logP	ACE IC_{50} (μM)
β-LGB	VAGTWYSLAMAASDISL	878,4	38,0	91,9
	VEELKPT	815,4	23,5	62,7
	VEELKPTPEGD	607,3	60,8	12,1
	VEELKPTPEGDL	663,8	51,7	584,9
	VEELKPTPEGDLE	728,4	56,6	411,5
	VEELKPTPEGDLEI	784,9	48,9	201,5
	VEELKPTPEGDLEIL	841,4	36,5	475,8
	VLDTDY	725,3	36,6	685,2
	VLVLDTD	774,4	32,7	55,0
	VLVLDTDY	937,5	49,8	685,2
	VRTPEVDD	930,5	32,9	203,3
	VRTPEVDDE	883,1	32,2	448,8
	VRTPEVDDEA	754,0	44,4	161,9
	VYVEELKPTPEGDLEI	916,0	55,2	201,5
	VYVEELKPTPEGDLEIL	972,5	48,6	475,8
	YVEELKPTPEGD	688,8	57,3	12,1
	YVEELKPTPEGDL	745,4	63,5	584,9
	YVEELKPTPEGDLE	809,9	48,4	411,5
	YVEELKPTPEGDLEI	866,4	39,3	201,5
	YVEELKPTPEGDLEIL	923,0	43,9	475,8
Pepsin	AIVDTGTS	763,4	38,4	131,2
	AIVDTGTSL	876,5	44,5	91,9
	DTVQVGGI	788,4	38,6	40,8
	FDNIWDQG	994,4	38,9	169,6
	FDNIWDQGLV	1005,7	51,0	976,1
	FDTGSSN	727,3	38,6	10,7
	FDTGSSNL	840,4	34,8	33,5
	FGLSETEPGS	1023,5	31,4	29,0
	FGTIGIGTPA	933,5	38,4	150,7
	GGISDTNQ	791,4	35,0	7,0
	GIGTPAQDF	905,4	41,6	162,9
	GILGYDTV	837,4	42,2	287,7
	GLSETEPGS	876,4	31,3	29,0
	GTGSMTGIL	836,4	26,5	475,8
	GTGSMTGILG	893,4	24,3	1219,6
	GTIGIGTPA	786,4	34,1	150,7
	GTIGIGTPAQD	1029,5	63,1	25,9
	GTPAQDF	735,3	42,2	162,9
	IGTPAQD	701,3	35,4	25,9
	QVGGISDTNQ	1018,5	59,7	7,0
	SIDSLPDI	859,4	26,8	684,1
	SIDSLPDIV	958,5	44,1	866,0
	SVEGYWQIT	1082,5	30,5	186,0

Protein	Sequence	m/z	-10logP	ACE IC_{50} (µM)
Pepsin	VDTGTSL	692,3	29,7	91,9
	VIFDTGSS	825,4	37,9	76,3
	WILGDV	702,4	26,5	1064,6
	YLDTEY	803,3	30,4	201,8
A0A140T8A9	IASGEPTSTPTIE	651,8	23,5	365,1
	STPTIE	647,3	26,7	365,1
	STPTIEA	718,4	24,2	161,9
A4IFI0	ISDFYPGSVT	1085,5	30,5	122,8
	SDFYPGSVT	972,4	36,6	122,8
	SVSGSLGQ	734,4	28,3	7,2
A5PJW4	ALVDTGTS	763,4	38,4	131,2
	WLLGDV	702,4	26,5	1064,6
E1BHU3	VIVLDTD	774,4	32,7	55,0
EPYC	DNNLDHIPLPL	630,8	35,4	160,3
	HIPLPL	689,4	24,5	160,3
	TDNNLDHIPLPL	681,4	52,4	160,3
F1MC90	LLVTQT	674,4	34,2	29,2
F1MH27	AIDLDDFTGT	1067,5	37,9	13,6
	SGAGPAGPY	776,4	37,4	187,8
F1MM81	AGPEAPFPGLP	1052,5	23,8	89,8
F1MWS3	VLFDTGSS	825,4	37,9	76,3
F1N177	GPFPLL	643,4	24,6	536,3
F1N4I1	STPTLE	647,3	26,7	411,5
G3N0E0	ALVDTGTSL	876,5	44,5	91,9
G3N0V0	SYMPEPVT	923,4	27,1	122,8
GLCM1	ILNKPEDET	1058,5	35,9	67,3
J9UHS4	HNSLPQ	695,3	26,4	33,3
	HNSLPQNIPPLTQT	780,4	49,7	29,2
	VYPFPGPIHN	1140,6	63,9	20,5
	VYPFPGPIHNSLPQ	783,4	59,3	33,3
Q28157	DSITMDGET	968,4	40,2	67,3
	DSITMDGETI	1081,5	31,5	184,9
	TLDSITMDGET	985,6	40,1	67,3
Q3MHH8	NVVDGQPF	875,4	38,3	44,6
Q5ZET1	EASPEVIESPPEINT	806,4	38,0	13,1
SPT22	NPELPPLTKT	1109,6	26,3	308,6
TKFC	SPAQLL	628,4	25,7	536,3

Protein	Sequence	m/z	-10logP	ACE IC_{50} (μM)
		F 60b I2		
β-casein	AVPYPQ	674,3	30,3	33,3
	DELQDKIHPF	621,3	36,4	44,6
	DMPIQ	619,3	27,3	98,8
	DMPIQ	603,3	28,7	98,8
	EAMAPK	662,3	34,2	422,2
	EELNVPGE	886,4	35,0	26,8
	EMPFPK	764,4	33,4	422,2
	EPVLGP	611,3	24,8	5,8
	FPPQSV	674,4	32,7	167,3
	GPFPII	643,4	26,3	556,4
	HKEMPFPK	844,8	38,4	422,2
	HLPLP	576,3	23,1	89,8
	HLPLPL	689,4	26,1	160,3
	HQPHQPLPPT	959,8	46,4	62,7
	HQPHQPLPPTVM	691,4	35,6	225,1
	KAVPYPQ	802,4	38,4	33,3
	KIHPF	641,4	29,4	44,6
	KVLPVPQ	780,5	28,6	33,3
	LHLPLP	689,4	25,4	89,8
	LHLPLPL	802,5	25,5	160,3
	LNVPGEIVE	969,5	31,5	241,0
	LTDVEN	690,3	30,6	20,1
	LTDVENL	803,4	29,8	33,5
	LTLTD	562,3	22,8	55,0
	LVYPFPGPI	1002,6	53,5	187,5
	LYQEPV	748,4	28,5	291,8
	LYQEPVLG	918,5	41,4	1219,6
	MHQPHQPLPPT	641,8	57,6	62,7
	MHQPHQPLPPTV	691,4	41,2	287,7
	MHQPHQPLPPTVM	756,9	48,9	225,1
	NIPPLTQT	883,5	43,8	29,2
	NLHLPLP	803,5	28,4	89,8
	NLHLPLPL	916,6	40,8	160,3
	NVPGEI	628,3	24,4	201,5
	NVPGEIVE	856,4	29,5	241,0
	PEVMGV	647,3	32,3	63,5
	PEVMGVS	734,3	37,2	260,7
	PFPGPIPN	838,4	41,2	18,7
	PFTESQ	708,3	29,5	19,1
	PFTESQS	795,4	37,8	61,9

Protein	Sequence	m/z	-10logP	ACE IC_{50} (µM)
β-casein	PGPIPN	594,3	22,8	18,7
	PPFLQPEV	926,5	24,7	313,5
	PQNIPPLT	879,5	28,2	209,7
	PQNIPPLTQT	1108,6	53,6	29,2
	PVEPF	588,3	27,1	44,6
	PVEPFT	689,4	35,0	256,5
	PVVVPPF	754,4	40,4	44,6
	PVVVPPFL	867,5	37,2	656,1
	PVVVPPFLQPE	814,8	40,0	123,0
	PVVVPPFLQPEV	660,9	59,3	313,5
	PVVVPPFLQPEVM	726,4	55,4	225,1
	PVVVPPFLQPEVM	734,4	65,8	225,1
	PVVVPPFLQPEVMG	762,9	67,7	915,2
	PVVVPPFLQPEVMGV	812,4	55,2	915,2
	PVVVPPFLQPEVMGVS	856,0	58,3	63,5
	PVVVPPFLQPEVMG	754,9	64,9	260,7
	PVVVPPFLQPEVMGVS	848,0	41,4	260,7
	QEPVLGPV	838,5	37,4	291,8
	QPLPPTVM	882,5	30,0	225,1
	SLPQNIPPLT	1079,6	34,5	209,7
	SLPQNIPPLTQT	654,9	44,9	29,2
	SLVYPFPGPIPN	650,9	31,5	18,7
	TDVENL	690,3	26,7	33,5
	TLTDVE	677,3	29,7	241,0
	TLTDVEN	791,4	43,4	20,1
	TLTDVENL	904,5	37,2	33,5
	TPVVVPPF	855,5	38,4	44,6
	TPVVVPPFLQPEV	711,4	36,5	313,5
	VENLHLPLPL	763,5	35,4	160,3
	VLPVPQ	652,4	22,7	33,3
	VPPFLQPEV	1025,6	32,0	313,5
	VPYPQ	603,3	26,9	33,3
	VVVPPFLQPEV	816,1	30,9	313,5
	VYPFPGPI	889,5	50,7	187,5
	VYPFPGPIPN	1100,6	60,4	18,7
	VYPFPGPIPNS	1187,6	65,9	27,8
	WMHQPHQPLPPT	742,9	38,3	48,7
	WMHQPH	835,4	28,9	62,7
	WMHQPHQPLPPT	734,9	62,0	62,7
	WMHQPHQPLPPTVM	849,9	60,5	225,1
	YPFPGPI	790,4	30,7	187,5
	YPVEPF	751,4	28,1	44,6
	YQEPV	635,3	25,8	291,8

Protein	Sequence	m/z	-10logP	ACE IC_{50} (µM)
β-casein	YQEPVLG	805,4	38,9	1219,6
	YQEPVLGPV	1001,5	46,4	291,8
αs1-casein	APFPEV	659,3	30,7	313,5
	AYFYPEL	902,4	33,1	172,3
	DAYPSGA	680,3	37,7	32,8
	EDVPSE	675,3	28,6	70,5
	EPMIGVN	775,4	28,8	63,5
	EPMIGV	645,3	27,7	36,7
	EPMIGVN	759,4	40,3	36,7
	FSDIPNPI	902,5	36,4	187,5
	FVAPFPEV	905,5	26,5	313,5
	HPIKH	631,4	24,8	239,6
	HQGLPQ	679,4	27,5	33,3
	HQGLPQE	808,4	34,9	57,2
	HQGLPQEV	907,5	47,1	313,5
	IQKEDVPSE	1044,5	32,4	70,5
	KEGIHA	654,4	25,2	165,0
	MKEGIHA	785,4	34,0	165,0
	QGLPQEV	770,4	33,3	313,5
	QKEPM	632,3	25,4	114,9
	QKEPMIGVN	1015,5	26,2	36,7
	QLDAYPS	793,4	27,2	133,0
	SDIPNPI	755,4	26,9	187,5
	SDIPNPIG	812,4	34,8	1081,9
	SDIPNPIGSEN	1142,5	52,3	20,1
	SDIPNPIGSENSEK	743,9	54,6	453,6
	SFSDIPNPI	989,5	49,2	187,5
	VAPFPEV	758,4	41,3	313,5
	VAPFPEVFG	962,5	43,1	1492,1
	VPLGTQ	614,3	29,2	32,8
	YFYPEL	831,4	30,1	172,3
	YQLDAYPS	956,4	39,6	133,0
	YQLDAYPSGA	1084,5	44,8	32,8
	YTDAPS	653,3	36,6	133,0
	YTDAPSF	800,3	36,2	25,6
	YTDAPSFSDIPNPI	768,9	39,2	187,5
αs2-casein	AINPSKENL	985,5	40,8	33,5
	ITVDDK	690,4	32,2	1540,3
	ITVDDKH	827,4	36,1	239,6
	LYQGPIV	789,4	31,8	866,0
	NAVPITPT	812,5	39,6	62,7
	NPWDQV	758,3	27,7	135,8
	VKITVDDK	917,5	39,2	1540,3

Protein	Sequence	m/z	-10logP	ACE IC_{50} (µM)
αs2-casein	YQGPIV	676,4	29,8	866,0
	YQGPIVLN	903,5	33,7	62,7
	YQKFPQ	810,4	29,8	33,3
β-LGB	EELKPTPEGDLE	678,8	35,9	411,5
	EKFDK	666,3	28,6	1540,3
	EKFDKA	737,4	26,2	742,0
	ELKPTPEGD	985,5	43,9	12,1
	ELKPTPEGDL	1098,6	47,5	584,9
	ELKPTPEGDLE	614,3	35,2	411,5
	ELKPTPEGDLEI	670,8	38,1	201,5
	GLDIQK	673,4	24,1	196,4
	GLDIQKVAGTWYSLAMAASDI	1105,6	34,1	684,1
	IDALNE	674,3	27,3	25,7
	ISLLDAQSAPL	1127,6	33,1	160,3
	IVTQT	561,3	22,0	29,2
	IVTQTM	692,4	34,2	113,3
	KIDALNE	802,4	40,5	25,7
	KPTPEGDL	856,4	34,9	584,9
	LIVTQTM	805,5	45,9	113,3
	LIVTQTM	821,4	39,8	113,3
	LIVTQTMK	933,5	52,5	1059,7
	LKPTPEGDL	969,5	41,1	584,9
	LKPTPEGDLE	915,6	32,2	411,5
	LLDAQSAPL	927,5	28,5	160,3
	LVLDTDY	838,4	35,7	685,2
	MAASDI	607,3	23,0	684,1
	PTPEGDL	728,3	27,3	584,9
	SFNPTQ	693,3	28,3	32,8
	SLAMAASD	765,3	36,9	32,0
	SLAMAASDI	878,4	46,7	684,1
	SLAMAASDIS	965,5	43,2	394,8
	SLAMAASDISLLDA	689,3	25,8	549,8
	SLAMAASDISLLDAQSAPL	937,5	35,7	160,3
	SLAMAASDISLLDAQSAPLR	1015,5	66,4	1267,9
	SLAMAASDISLLDAQSAPLRV	1065,1	61,3	2181,7
	TPEVDD	675,3	32,5	203,3
	TPEVDDEA	875,4	53,0	161,9
	TPEVDDEALEK	623,3	51,1	453,6
	TPEVDDEALEKFDK	818,4	42,5	1540,3
	VAGTWYSLAMAASDISL	878,4	29,8	91,9
	VAGTWYSLAMAASDISLL	935,0	43,6	536,3
	VAGTWYSLAMAASDISLLDA	1028,0	31,9	549,8
	VEELKPTPE	868,1	34,4	123,0

Protein	Sequence	m/z	-10logP	ACE IC_{50} (μM)
β-LGB	VEELKPTPEGD	607,3	51,1	12,1
	VEELKPTPEGDL	663,8	51,3	584,9
	VEELKPTPEGDLE	728,4	61,6	411,5
	VEELKPTPEGDLEI	784,9	51,6	201,5
	VLDTDY	725,3	36,1	685,2
	VLVLDT	659,4	27,4	228,7
	VLVLDTD	774,4	32,3	55,0
	VLVLDTDY	937,5	47,6	685,2
	VLVLDTDYK	710,7	33,1	828,7
	VRTPEVDD	930,5	36,7	203,3
	VRTPEVDDE	706,7	41,4	448,8
	VRTPEVDDEA	565,8	40,5	161,9
	VTQTMK	707,4	26,6	1059,7
	VYVEELKPTPEGDL	794,9	50,0	584,9
	VYVEELKPTPEGDLEI	916,0	43,9	201,5
	VYVEELKPTPEGDLEIL	972,5	42,1	475,8
	YVEELKPTPEGD	688,8	48,4	12,1
	YVEELKPTPEGDL	745,4	46,9	584,9
	YVEELKPTPEGDLE	809,9	30,7	411,5
	YVEELKPTPEGDLEI	866,4	39,9	201,5
	YVEELKPTPEGDLEIL	923,0	46,8	475,8
Pepsin	AIVDTGT	676,4	25,2	13,6
	AIVDTGTS	763,4	42,6	131,2
	AIVDTGTSL	876,5	40,4	91,9
	DTGSSNL	693,3	30,7	33,5
	DTVQVGGI	788,4	37,2	40,8
	EGYWQIT	896,4	26,3	186,0
	FDNIWDQG	994,4	37,5	169,6
	FDNIWDQGLV	804,7	42,9	976,1
	FDTGSS	613,2	27,8	76,3
	FDTGSSN	727,3	44,2	10,7
	FDTGSSNL	840,4	42,8	33,5
	FGLSETEPGS	1023,5	25,4	29,0
	FGTIGIGTPA	933,5	38,7	150,7
	GGISDTNQ	791,4	36,4	7,0
	GGISDTNQI	904,4	34,1	87,2
	GIGTPAQDF	905,4	40,7	162,9
	GLAYPSIS	807,4	31,6	394,8
	GLSETEPGS	876,4	26,0	29,0
	GTGSMTGILG	893,4	35,1	1219,6
	GTIGIGTPA	786,4	34,8	150,7
	GTIGIGTPAQD	1029,5	54,8	25,9
	GTIGIGTPAQDF	784,7	39,2	162,9

**Protein**	**Sequence**	**m/z**	**-10logP**	**ACE IC_{50} (µM)**
Pepsin	GTPAQDF	735,3	38,0	162,9
	IGTPAQD	701,3	36,9	25,9
	QVGGISDTN	890,4	53,1	18,5
	QVGGISDTNQ	1018,5	57,5	7,0
	SIDSLPDI	859,4	31,6	684,1
	SIDSLPDIV	958,5	49,0	866,0
	TVIFDTGSS	926,5	43,5	76,3
	VDTGTSL	692,3	26,9	91,9
	VGGISDTNQ	890,4	43,9	7,0
	VIFDTGSS	825,4	36,1	76,3
	YAPFDGILG	952,5	29,1	1219,6
A0A0M3R7C5	EDSPEVIESPPEINT	828,4	35,3	13,1
	LEDSPEV	788,4	30,6	313,5
	TLEDSPEV	889,4	26,3	313,5
A0A140T8A9	EQNQEQPI	985,5	33,7	187,5
	KYIPIQ	761,5	24,0	98,8
A1A4J4	ITLTD	562,3	22,8	55,0
A5PJW4	ALVDTGT	676,4	25,2	13,6
	ALVDTGTS	763,4	42,6	131,2
E1B9Y2	ITITD	562,3	22,8	55,0
E1BEZ1	SPAQLL	628,4	24,7	536,3
E1BHU3	VIVLDT	659,4	27,4	228,7
	VIVLDTD	774,4	32,3	55,0
E1BJE0	LVTQT	561,3	22,0	29,2
E7E1Q1	STPTIE	647,3	28,2	365,1
	STPTIEA	718,4	26,7	161,9
EPYC	DNNLDHIPLPL	630,8	38,6	160,3
	HIPLP	576,3	23,1	89,8
	HIPLPL	689,4	26,1	160,3
	TDNNLDHIPLPL	681,4	48,0	160,3
F1ME93	KLHPF	641,4	29,4	44,6
F1MH27	AIDLDDFTGT	1067,5	46,7	13,6
	NFGTAPF	753,4	27,8	44,6
	SGAGPAGPY	776,4	44,5	187,8
F1MLW7	ISDFYPGSVT	1085,5	35,6	122,8
	SDFYPGSVT	972,4	36,2	122,8
	SVSGSLGQ	734,4	27,2	7,2
F1MPA5	DTGSSNI	693,3	30,7	39,2
F1MWS3	VLFDTGSS	825,4	36,1	76,3
F1N440	HLPIP	576,3	23,1	79,7
G3N2I5	GPFPLL	643,4	26,3	536,3
G5E533	LDAINE	674,3	27,3	25,7
GLCM1	ILNKPEDET	1058,5	34,4	67,3

Protein	Sequence	m/z	-10logP	ACE IC_{50} (µM)
J9UHS4	HNSLPQ	695,3	33,2	33,3
	HNSLPQNIPPLTQT	780,4	47,2	29,2
	VYPFPGPIHN	1140,6	52,0	20,5
	VYPFPGPIHNSLPQ	783,4	53,6	33,3
PD2R	LTITD	562,3	22,8	55,0
Q28049	DDDLTDDIM	1052,4	39,7	341,0
	DLTDDIM	822,4	31,1	341,0
	FLDDDLTD	953,4	28,6	55,0
	FLDDDLTDD	1068,4	41,6	203,3
	GGVSLPEW	844,4	25,2	23,3
	GGVSLPEWV	943,5	32,8	1902,0
	ILDKVGIN	871,5	27,0	55,6
	IVQNNDSTE	1019,5	37,9	121,3
	LDDDLTDD	921,4	37,6	203,3
	LDDDLTDDI	1034,5	32,8	684,1
	LDDDLTDDIM	1165,5	45,2	341,0
Q28157	DSITMDGET	968,4	41,8	67,3
	DSITMDGETI	1081,5	31,4	184,9
	DSITMDGETIA	1152,5	33,1	447,2
Q2TA20	NPELPPLTKT	1109,6	28,4	308,6
Q3MHH8	ALVFVDNHDNQR	714,4	41,1	176,4
	IYVDAV	679,4	27,2	266,3
	NVVDGQPF	875,4	38,9	44,6
	SISNSAEDPF	1066,5	40,1	44,6
Q5ZET1	ARHPHPH	851,4	32,8	48,7
	KTEIPTINT	1016,6	34,2	13,1
	YQQKPVA	833,5	30,8	295,3
	EASPEVIESPPEIN	755,9	31,6	55,6
	EIPTINT	787,4	38,6	13,1
	ESPPEIN	785,4	37,0	55,6
	ESPPEINT	886,4	46,7	13,1
	ESPPEINTV	985,5	32,5	287,7
	FLPYPY	799,4	25,0	187,8
	HPHPH	624,3	30,0	48,7
	IASGEPTSTPT	1060,5	34,9	62,7
	IASGEPTSTPTTE	645,8	32,9	121,3
	IESPPEIN	898,5	43,6	55,6
	IESPPEINT	999,5	51,9	13,1
	LPYPY	652,3	24,0	187,8
	MAIPPK	656,4	26,8	422,2
	NQDKTEIPT	871,4	26,4	62,7
	NQDKTEIPTINT	687,3	38,3	13,1
	QFLPYPY	927,5	30,4	187,8

Protein	Sequence	m/z	-10logP	ACE IC_{50} (µM)
Q5ZET1	SPAQIL	628,4	24,7	475,8
	SPAQILQ	756,4	28,8	111,4
	TEIPTIN	787,4	36,7	55,6
	TEIPTINT	888,5	47,3	13,1
	TIASGEPT	775,4	25,7	62,7
	VIESPPEIN	997,5	39,3	55,6
	VQVTSTAV	804,4	43,0	266,3
	YPSYGLN	813,4	40,2	62,7
	YYQQKPVA	996,5	34,1	295,3
Q85PG9	TVLFDTGSS	926,5	43,5	76,3
Q9TTV8	ALVDTGTSL	876,5	40,4	91,9
